# The *Bursaphelenchus xylophilus* effector BxML1 targets the cyclophilin protein (CyP) to promote parasitism and virulence in pine

**DOI:** 10.1186/s12870-022-03567-z

**Published:** 2022-04-27

**Authors:** Yan Zhang, Tong-Yue Wen, Xiao-Qin Wu, Long-Jiao Hu, Yi-Jun Qiu, Lin Rui

**Affiliations:** 1 Co-Innovation Center for Sustainable Forestry in Southern China, College of Forestry, Nanjing Forestry University, Nanjing, 210037 China; 2 Jiangsu Key Laboratory for Prevention and Management of Invasive Species, Nanjing Forestry University, Nanjing, 210037 China

**Keywords:** *Bursaphelenchus xylophilus*, Effector, MD-2-related lipid-recognition domain protein, Cyclophilin protein, *Pinus thunbergii*

## Abstract

**Background:**

*Bursaphelenchus xylophilus* is the causal agent of pine wilt disease (PWD) that has caused enormous ecological and economic losses in China. The mechanism in the interaction between nematodes and pine remains unclear. Plant parasitic nematodes (PPNs) secrete effectors into host plant tissues. However, it is poorly studied that role of effector in the infection of pine wood nematode (PWN).

**Results:**

We cloned, characterized and functionally validated the *B. xylophilus* effector BxML1, containing an MD-2-related lipid-recognition (ML) domain. This protein inhibits immune responses triggered by the molecular pattern BxCDP1 of *B. xylophilus*. An insitu hybridization assay demonstrated that BxML1 was expressed mainly in the dorsal glands and intestine of *B. xylophilus*. Subcellular localization analysis showed the presence of BxML1 in the cytoplasm and nucleus. Furthermore, number of *B. xylophilus* and morbidity of pine were significantly reduced in *Pinus thunbergii* infected with *B. xylophilus* when BxML was silenced. Using yeast two-hybrid (Y2H) and coimmunoprecipitation (CoIP) assays, we found that the BxML1 interacts with cyclophilin protein PtCyP1 in *P. thunbergii*.

**Conclusions:**

This study illustrated that BxML1 plays a critical role in the *B. xylophilus*–plant interaction and virulence of *B. xylophilus.*

**Supplementary Information:**

The online version contains supplementary material available at 10.1186/s12870-022-03567-z.

## Introduction

Pine wilt disease (PWD) is one of the most destructive diseases of pine trees and is currently a worldwide issue [1]. *Bursaphelenchus xylophilus* caused PWD from North America and spread to Japan in the early twentieth century [2, 3]. In Europe, it was first reported in Portugal in 1999 [4]. China was first discovered PWD in Purple Mountain of Nanjing City in 1982 in Jiangsu Province [5]. In 2021, the PWD outbreak area in mainland China reached 1.8092 hm, and the number of diseased and dead pine trees was 19,470,300 [6]. *B. xylophilus* has a complex life cycle witch including two different stages: the phytophagous parasitic stage and the mycophagous stage [7]. In the phytophagous parasitic stage, a strong relationship between *B. xylophilus* and pine is established, *B. xylophilus* can rapidly reproduce and spread in pine trees and destroy tissues of the cortex, phloem, cambium and resin canals [8]. As the trees die, the *B. xylophilus* turns to the mycophagous stage, migrating to beetle pupal chambers and feeding on fungi in dead or dying trees, then being transmitted by the insect vector *Monochamus* spp. to healthy pine trees [9]. However, the mechanism of *B. xylophilus*–plant interactions remains elusive.

To facilitate parasitism, plant-parasitic nematodes (PPNs) secrete effector proteins into plants via the stylet in their mouth parts. These effectors play vital roles in the infection stages [10]. Plants have also evolved multiple immune responses to pathogens. The first layer of defence is triggered by the recognition of pathogen-associated molecular patterns (PAMPs) by transmembrane pattern recognition receptors (PRRs); this defence is often referred to as PAMP-triggered immunity (PTI) or basal defence [11]. In turn, pathogens have evolved effector molecules to cope with PTI in host plants. Plants can also perceive pathogen effectors intracellularly using nucleotide-binding site leucine-rich repeat immune receptors. These receptors recognize plant pathogen molecules to activate effector-triggered immunity (ETI) [12]. PTI and ETI are forms of innate immunity that synergistically activate plant defence responses to prevent invasion by microorganisms [13]. PPNs deploy various effectors to suppress PTI and ETI during interactions with plants [14, 15]. Molecular research related to PPNs has mainly focused on two groups of sedentary endoparasitic nematodes: root knot nematodes (RKNs, *Meloidogyne* spp.) and cyst nematodes (CNs, *Globodera* and *Heterodera* spp.) [16]. PPN effector proteins are diverse and can trigger a variety of plant immune responses [16]. For example, NILR1 is a leucine-rich repeat receptor-like kinase, which is the first example of an immune receptor involved in induction of basal immunity (PTI) in plants or in animals in response to nematodes [17]. Some effectors can suppress plant defences and promote parasitism. For instance, the *H. schachtii* effector Hs4E02 can inhibit plant immunity by targeting and repositioning the host hemilysine protease RD21a, which plays an important role in the plant defence response [18].

For *B. xylophilus*, BxCDP1 is recognized as a novel molecular pattern [19]. In addition, a series of effectors that trigger immunity were screened out (BxSapB1, BxSapB2, BxSapB3) [20–22]. Recent research on *B. xylophilus* effectors has led to the characterization of some effectors related to inhibiting host defence. For example, the PWN effector Bx-FAR-1 has a fatty acid- and retinol-binding domain, and BxSCD1 can inhibit plant immunity and interact with the ethylene formation enzyme in pine trees [23, 24]. It was reported that RKNs and CNs are predicted to secrete hundreds of effectors [16]. However, little is known about the functional characteristics of more *B. xylophilus* effectors, especially that inhibiting host defence. Further characterization of *B. xylophilus* effectors is therefore essential to explore the mechanisms underlying the pine–*B. xylophilus* interaction and for management of PWD.

ML proteins are defined as proteins with an MD-2-related lipid-recognition domain and are ubiquitously found in bacteria, fungi, plants, and animals. MD-2 consists of two antiparallel β-sheets that form a hydrophilic pocket [25–27]. These proteins were predicted to mediate diverse biological functions by interacting with specific lipids, such as lipopolysaccharide ligands, which are recognized as PAMPs that induce innate immune responses in mammals [28]. Encoded by the Niemann-Pick Type C2 (NPC2) gene, the NPC2 protein is another well-characterized ML protein. Mutations disrupting the functioning of NPC2 proteins cause a lipid storage disorder called Niemann Pick disease Type C, characterized by cholesterol accumulation in lysosomes [11].

*B. xylophilus* ML protein (BxML1) was identified by transcriptomic analysis as being upregulated in the infection stage [29]. The biochemical and cell biological functions of *B. xylophilus* ML proteins, especially in the interactions with plants, are completely unknown. In the present study, we explored the mechanism by which BxML1 affects plant PTI responses and the expression patterns of BxML1. Via RNA interference (RNAi), we verified the crucial roles of BxML1 in the feeding and reproduction of *B. xylophilus* in the mycophagous stage and in infection in the phytophagous parasitic stage. In addition, yeast two-hybrid (Y2H) experiments verified the interaction of BxML1 with pine cyclophilin protein (CyP).

## Results

### Sequence analysis of the BxML1 gene from *B. xylophilus*

**Fig. 1:**
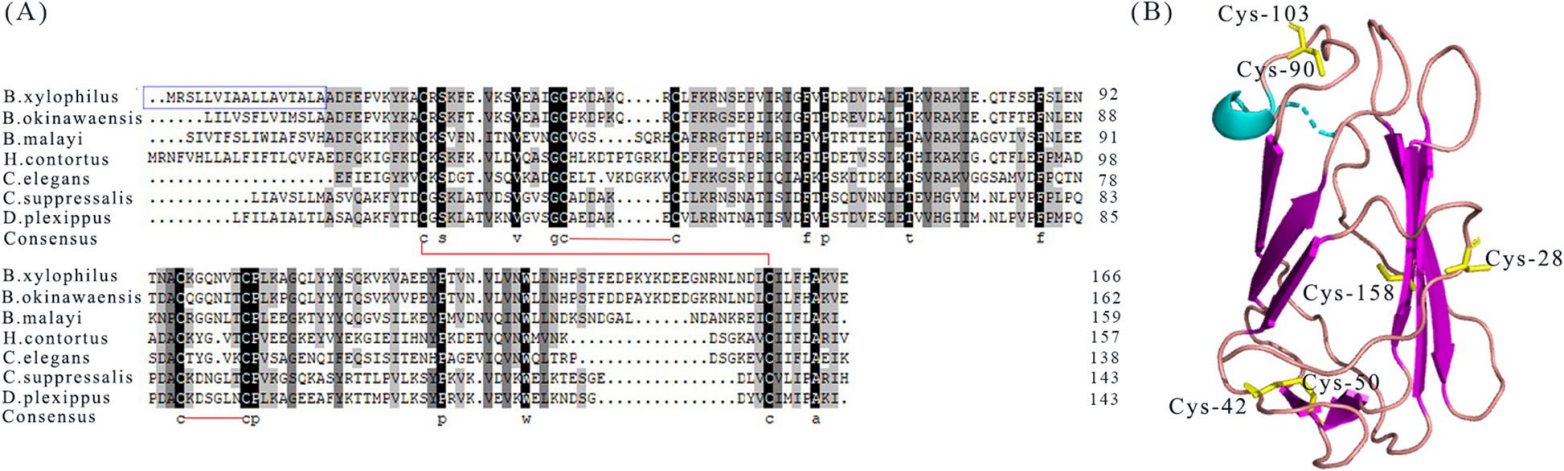
Primary sequence alignments of BxML1 orthologs and related proteins. (**A**) Multiple sequence alignment of the predicted BxML1 protein with ML from different species. The blue box indicates the predicted SP of BxML1, Conserved residues are highlighted in gray, with identical residues printed in black, Red lines connect cysteine residues that form disulfide bonds. (**B**) Structure of BxML1. β-sheet is shown in red. The side chains of cysteine residues that form three disulfide bonds are shown in yellow

To further study the conservation and structural characteristics of BxML1, we performed sequence alignment and predicted the 3D structure. BxML1 had a length of 504 bp (Fig. S1) and encoded a 162-amino-acid protein with a molecular weight of 19.81 kD and a pI of 8.16. According to the NCBI Conserved Domain Database, the putative translation product of BxML1 included an MD-2-related lipid-recognition (ML) domain. Multiple sequence alignment of the protein showed that BxML1 was characterized by six conserved cysteines. The protein encoded by the BxML1 gene shared approximately 80.25% identity and 97% similarity with an unannotated protein in *Bursaphelenchus okinawaensis* and shared 41.21% identity and 97% similarity with an ML protein from *Brugia malayi* (Fig. 1A). Most of these orthologous proteins had predicted N-terminal signal peptides (SPs), which is consistent with the characteristics of ML proteins. The three-dimensional construct of BxML1 comprise conserved cysteine residues and four antiparallel *β*-strands is also consistent with the characteristics of ML proteins (Fig. 1B).

### BxML1 suppresses BxCDP1-induced cell death in *N. benthamiana*

**Fig. 2:**
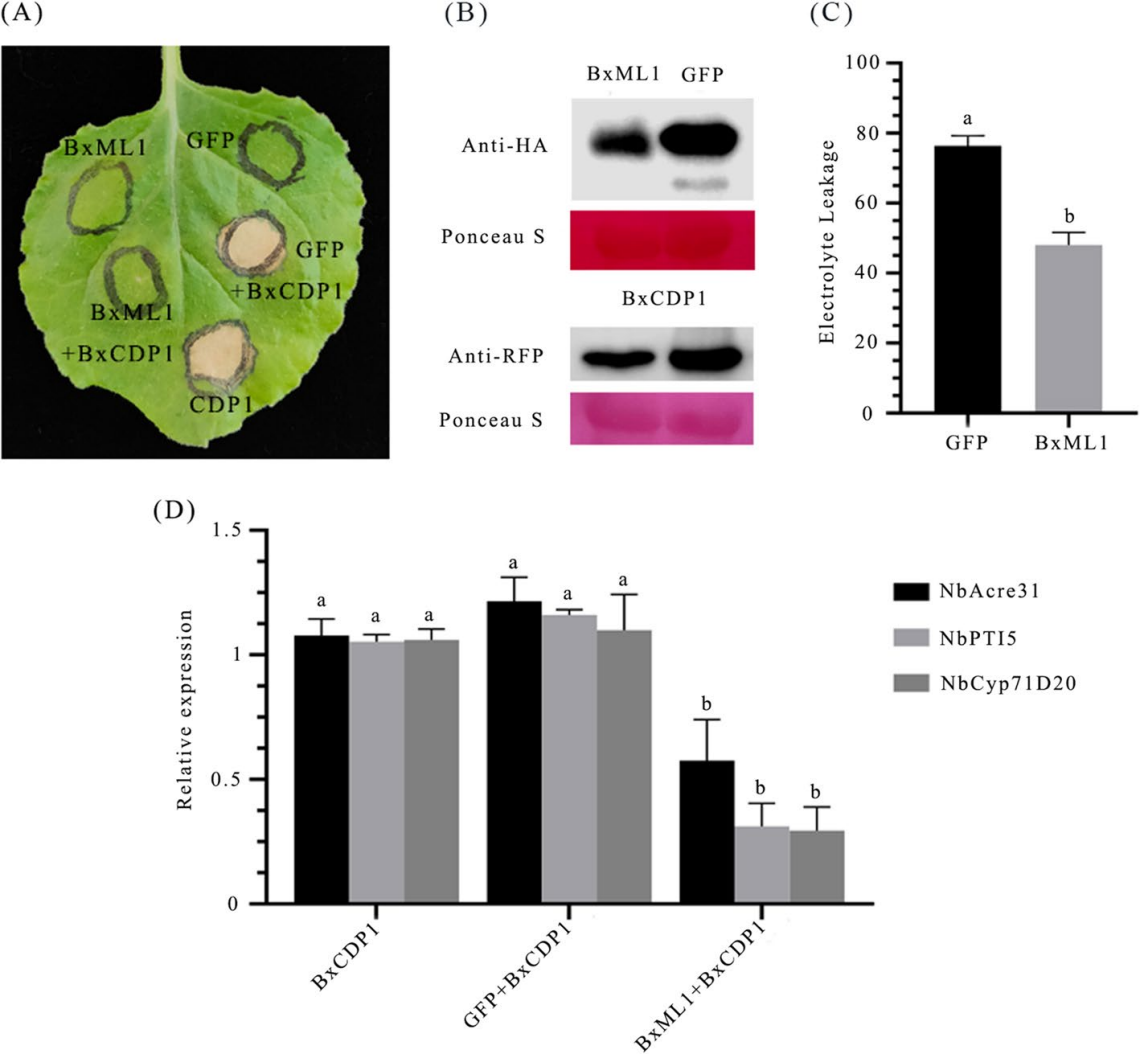
BxML1 suppresses immunity-associated hypersensitive cell death in *Nicotiana benthamiana*. (**A**) Coexpression of BxML1^Δsp^ (right of leaf) suppressed BxCDP1-triggered hypersensitive cell death in *N. benthamiana*. Pictures were taken 6 days postinfiltration. The experiments were repeated at least three times with similar results. (**B**) Immunoblot analysis of proteins from *N. benthamiana* leaves transiently expressing target proteins. Protein loading is indicated by Ponceau S staining of RuBisCO (This picture has been cropped. For full length original blot, see Additional file 7). (**C**) Quantification of suppression of cell death by measuring electrolyte leakage in *N. benthamiana*. The data shown are combined from three independent experiments. Different letters over error bars indicate statistically significant differences using Duncan’s multiple range test (*p* < 0.05). (**D**) Transcript level of NbAcre31, NbPTI5, and NbCyp71D20 induced by 300 nM BxCDP1 in *N*. *benthamiana* leaf tissue expressing the BxML1 and GFP proteins. Values represent the mean ± *SD* of three independent biological samples. Different letters over error bars indicate statistically significant differences using Duncan’s multiple range test (*p* < 0.05)

We performed *Agrobacterium tumefaciens*-mediated transient expression of BxML1 in *N.benthamiana* leaves to investigate the influence of BxML1 on plant immunity. As a novel molecular pattern of *B. xylophilus*, BxCDP1 can trigger programmed cell death in many plants [19]. Agrobacteria carrying BxML1 were infiltrated into *N*. *benthamiana* leaves 16 h before infiltration with Agrobacterium strains carrying BxCDP1, and green fluorescent protein (GFP) was used as a control. We found that BxCDP1-triggered programmed cell death (PCD) was suppressed at 6 days post inoculation (dpi) (Fig. 2A). We verified the expression of each of the relevant proteins by immunoblotting (Fig. 2B). In addition, electrolyte leakage in *N. benthamiana* triggered by BxCDP1 was markedly weakened in the presence of BxML1 (Fig. 2C). A previous study demonstrated that PTI marker genes (NbAcre31, NbPTI5, and NbCyp71D20) were upregulated by treatment with BxCDP1 in response to biotic stresses [19]. Therefore, we infiltrated purified BxCDP1 protein into *N*. *benthamiana* leaves 16 h after expressing the BxML1 or GFP (negative control) protein. Quantitative reverse transcription PCR (RT-qPCR) analysis revealed that BxML1 reduced the expression level of NbAcre31, NbPTI5, and NbCyp71D20 (Fig. 2D). These results suggest that BxML1 can suppress BxCDP1-triggered cell death.

### BxML1 is predominantly expressed in the dorsal glands and intestine and upregulated during the early stages of pine infection

**Fig. 3:**
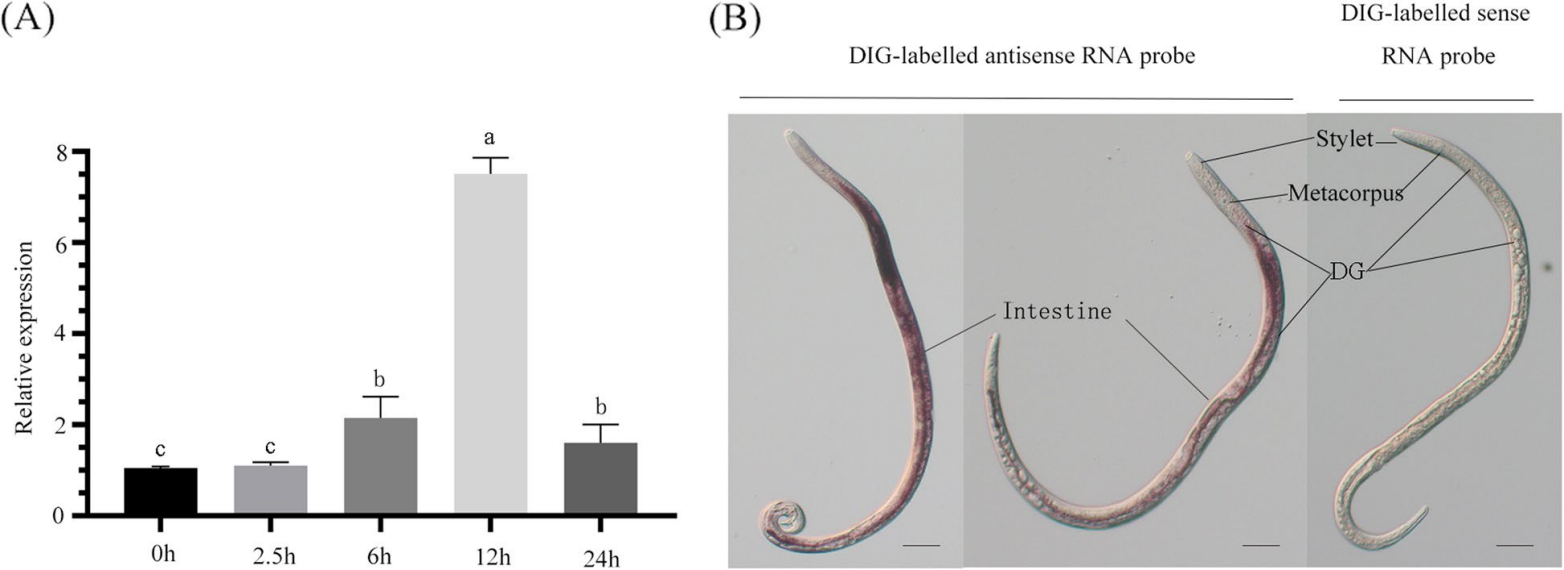
Expression pattern of BxML. (**A**) The expression level of BxML1 at the early stages of *Bursaphelenchus xylophilus* infection by quantitative reverse transcription PCR (RT-qPCR) analysis. *P. thunbergii* seedlings inoculated with *B. xylophilus* (AMA3) were sampled at different time points. The relative transcript level of BxML1 was calculated by the comparative threshold method. The RT-qPCR values were normalized to the transcript level of Actin. Values represent the mean ± SD of three independent biological samples. Different letters over error bars indicate statistically significant differences using Duncan’s multiple range test (*p <* 0.05). (**B**) Localization of BxML1 in the dorsal glands (DG) by in situ hybridization. DIG, digoxygenin; M, median bulb. Scale bars = 20 μm

In general, effector genes are transcriptionally upregulated during the early infection stages [30]. The expression of BxML1 at 0 h, 2.5 h, 6 h, 12 h and 24 h after infestation was quantified by RT-qPCR. The expression levels of BxML1 tended to stabilize in the mycophagous stage and within 6 h after infection. However, the relative expression levels of BxML1 in the phytophagous stage were 7 times higher than those in the mycophagous stage (Fig. 3A). This finding indicated that BxML1 was abundantly expressed in the early stage of *B. xylophilus* infection.

The tissue localization of BxML1 transcripts in the nematode was verified using in situ hybridization. We collected PWNs from whole seedlings using the Baermann funnel technique 12 h after inoculation into pine stems. A DIG-labelled BxML1 antisense complementary DNA (cDNA) probe was used. The results demonstrated that *BxML1* was specifically expressed in the dorsal glands (DG) and intestines of PWNs, but no signal was detected when a sense cDNA probe was used (Fig. 3B, S4)*.* This finding indicated that BxML1 could be secreted into plant tissues and plays a vital role during the early stages of *B. xylophilus* infection of pine.

### Subcellular localization of BxML1 in *N. benthamiana* cells

**Fig. 4:**
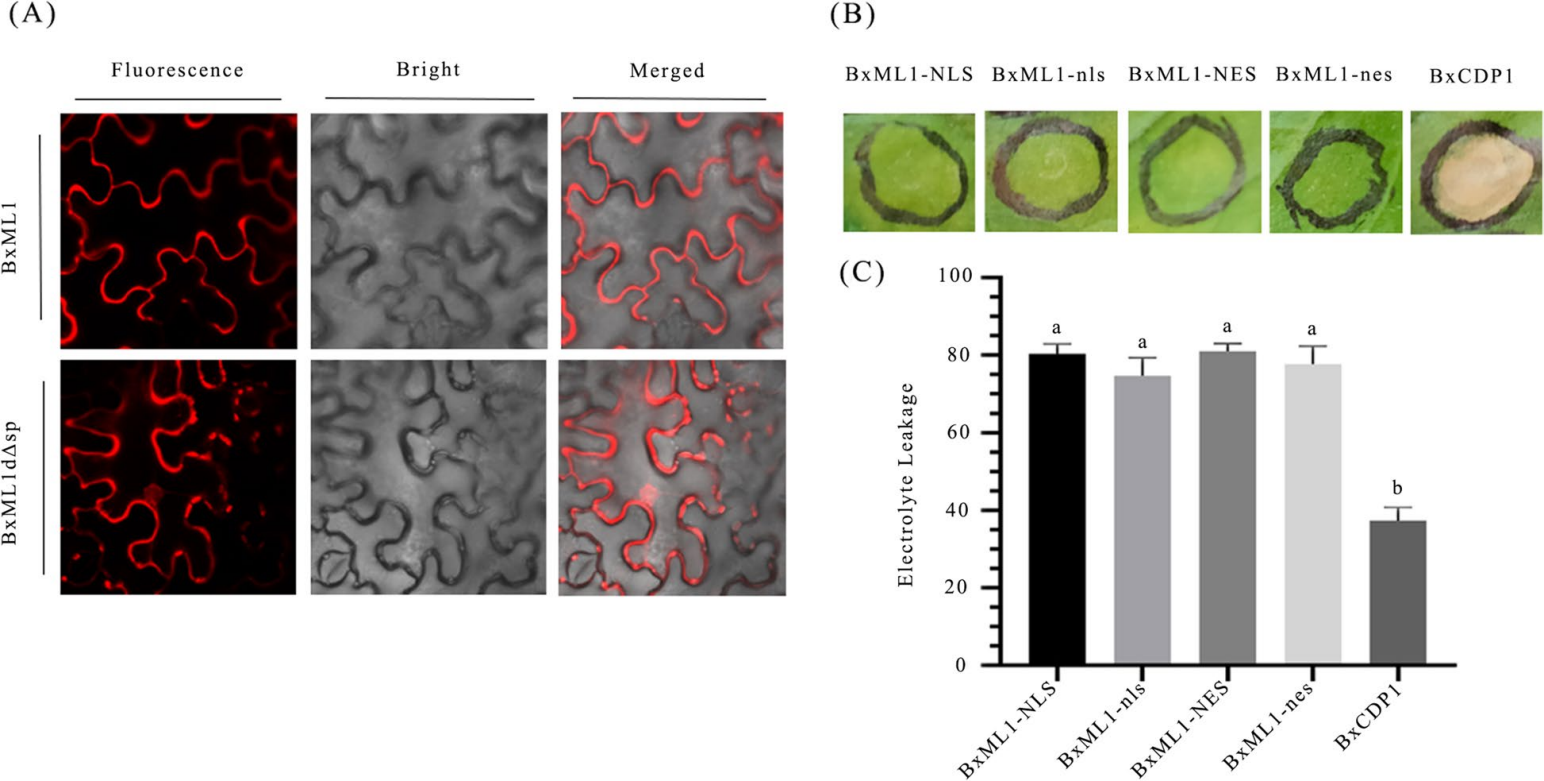
BxML1 is located in the cytoplasm and nucleus and not dependent on a particular cellular localization for suppression of BxCDP1-induced cell death (**A**) Subcellular localization of BxML1 was determined by transient expression of red fluorescent protein (RFP)-tagged proteins in *N. benthamiana* leaves. (**B**) BxML1-NES and BxML1-NLS suppresses BxCDP1-induced cell death in *N. benthamiana* leaves. Pictures were taken 5 days postinfiltration. The experiments were repeated at least three times with similar results. (**C**) Quantification of suppression of cell death by measuring electrolyte leakage in *N. benthamiana*. The data shown are combined from three independent experiments. Different letters over error bars indicate statistically significant differences using Duncan’s multiple range test (*p* < 0.05)

To investigate the subcellular localization of BxML1 in plant cells, a vector harbouring red fluorescent protein (RFP)-tagged BxML1 (without an SP) was constructed, and *N. benthamiana* leaves were used to perform a transient expression assay. The results showed that BxML1 was primarily accumulated in the cytoplasm and nucleus in *N. benthamiana* (Fig. 4A)*.*

Most effectors exhibit nuclear localization. To assess whether the nuclear localization of BxML1 is required for the suppression of cell death, we fused a nuclear export signal (NES), a mutated form of the NES (nes), a nuclear localization signal (NLS) or a mutated form of the NLS (nls) to BxML1. When these BxML1 mutants were expressed in *N*. *benthamiana*, they all suppressed BxCDP1-induced cell death (Fig. 4B), electrolyte leakage in *N. benthamiana* triggered by BxCDP1 was markedly weakened in the presence of BxML1 (Fig. 4C). indicating that the cellular localization was not restricted to the cytoplasm or nucleus.

### BxML1 contributes to the fecundity and virulence of *B. xylophilus*

**Fig. 5:**
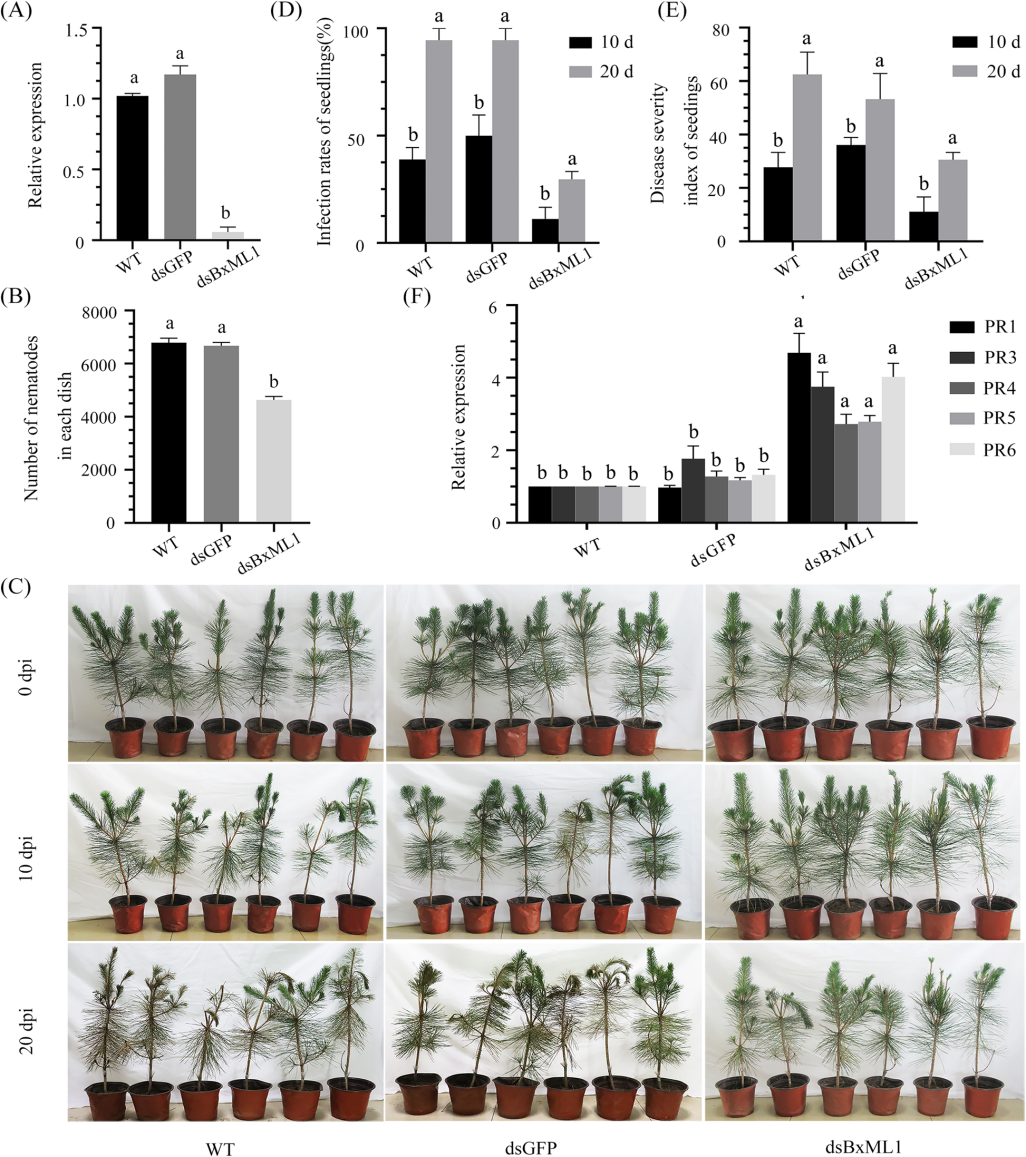
RNAi of BxML1 affects feeding and reproduction and parasitism of *P. thunbergia* of pine wood nematode. (**A**) The silencing efficiency of BxML1 in *B. xylophilus* (**B**) The number of nematodes on *B. cinerea* over 7 days, each dish of *B. cinerea* was inoculated with 80 *B. xylophilus* (40 female nematodes and 40 male nematodes), Data are presented as the means ± standard deviation (SD) from three experiments. Different letters indicate significant differences (*P* < 0.05). (**C**) The symptoms of *P. thunbergii* at 10and 20 days post-inoculation (dpi) with three different nematode treatments (WT, dsGFP, and dsBxML1). (**D**-**E**) The infection ratio and disease severity index of pine seedlings were calculated at 10 and 20 dpi. Three independent experiments were performed, and 13 individual *P. thunbergii* seedlings were used for each treatment. Data are presented as the means ± standard deviation (SD) from three experiments. Different letters indicate significant differences (*P <* 0.05) (**F**) Relative expression levels of pathogenesis-related gene in *P. thunbergii* infected with dsRNA-treated nematodes. At 12 hpi, the *P. thunbergii* stem samples were collected to analyze the change in the relative expression of PR1, Data are presented as the means ± standard deviation (SD) from three biological replicates. Different letters indicate significant differences (*P <* 0.05)

We used RNAi to investigate the roles of BxML1 in the fecundity, feeding and virulence of *B. xylophilus*. PWNs were soaked in BxML1 or GFP dsRNA for 48 h at 20 °C in the dark for RNAi. The expression of BxML1 in different treatment groups was subsequently measured by RT-qPCR. The results showed that BxML1 expression decreased significantly after soaking in BxML1 dsRNA compared to that after soaking in GFP dsRNA and that in the wild type (WT) (Fig. 5A). The *Botrytis cinerea* mycelia on the potato dextrose agar (PDA) plates used for culturing the PWNs and the number of PWNs on the *B. cinerea* plates were counted. The dsBxML1-treated nematodes showed a significant delay in feeding and fecundity (Fig. 5B, S2). Moreover, the appearance of symptoms in *Pinus thunbergii* was delayed after infection with dsBxML1-treated PWNs. Pine infected with dsBxML1-treated PWNs remained healthy, while pine infected with dsGFP-treated and WT PWNs exhibited distinct yellow needles at 10 dpi. The appearance of symptoms in pine infected with dsBxML1-treated PWNs occurred after 20 dpi (Fig. 5C). The infection rates and disease severity index were calculated at 10 and 20 dpi (Fig. 5D, E). These results suggested that BxML1 contributes to fecundity, feeding and infection in *B. xylophilus*.

We further evaluated whether BxML1 silencing in *B. xylophilus* influences the pine defence response. We analysed the fold change in gene expression of pathogenesis-related proteins (PRs) 12 h after PWN inoculation using RT-qPCR (Fig. 5F). The defence marker gene PR1 was expressed at a much higher level in pine infected with dsBxML1-treated PWNs than in pine infected with dsGFP-treated and WT PWNs. In conclusion, the RNAi data of BxML1 showed that BxML1 is important for the parasitism and virulence of *B. xylophilus*.

### BxML1 specifically interacts with the *P. thunbergii* cyclophilin protein PtCyP1

**Fig. 6:**
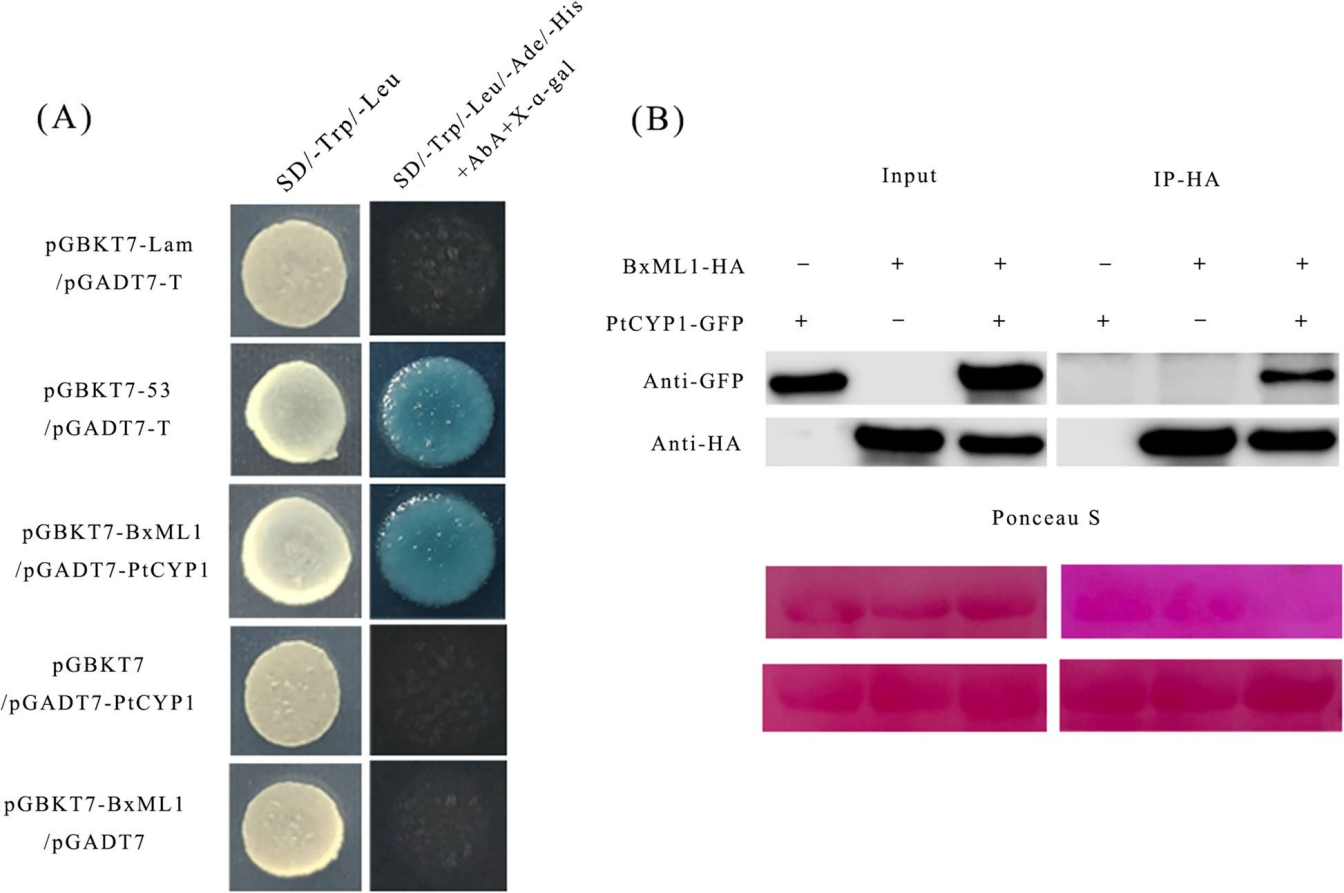
BxML1 interacts with *P. thunbergia* CyP1 proteins. (**A**) BxML1 interacts with PtCyP1 in yeast. Yeast strain Y2H Gold co-carrying BD-BxML1 and AD-PtCyP1 was grown on SD/−Trp/−Leu and the selective medium SD/−Trp/−Leu/−His/−Ade /X-α-Gal/AbA. (**B**) BxML1 interacts with PtCyP1 in vivo. Coimmunoprecipitation (IP) was performed on extracts of *N. benthamiana* leaves expressing both BxML1-HA and PtCyP1-GFP. Green fluorescent protein (GFP) was detected by western blot using anti-GFP antibodies. HA was detected by western blot using anti-HA antibodies. The immune complexes were pulled down using anti-HA agarose beads (This picture has been cropped. For full length original blot, see Additional file 7)

To elucidate the molecular mechanism underlying BxML1-mediated suppression of host immunity, we screened the potential *P. thunbergii* targets of BxML1 by using a Y2H assay. A *P. thunbergii* cyclophilin protein (PtCyP1) was originally identified as a candidate interaction partner of BxML1 by a Y2H screen. Subsequently, we confirmed the interaction between full-length BxML1 and PtCyP1 in yeast. The interaction between full-length BxML1 and PtCyP1 was demonstrated by Y2H (Fig. 6A). This interaction was further confirmed by a Co-IP assay (Fig. 6B). The above results show that BxML1 specifically interacts with the *P. thunbergii* cyclophilin protein in the process of the interaction between *B. xylophilus* and pine.

### BxML1 inhibits the expression of PtCyP1 in *P. thunbergii*

**Fig. 7:**
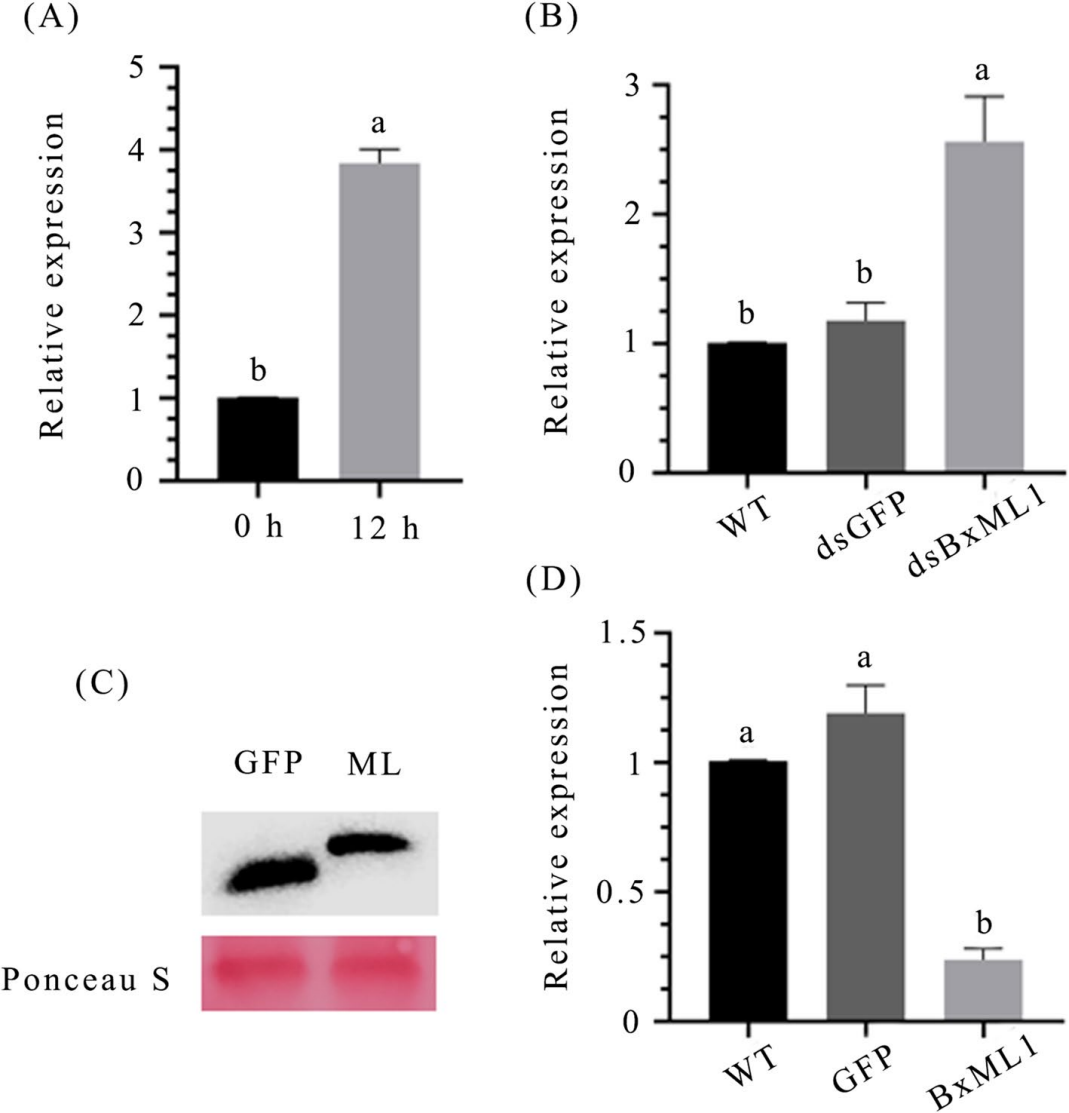
(**A**) PtCyP1 is up-regulated in *Pinus sylvestris* during nematode infestation at 0 and 12 h, the *P. thunbergii* stem samples were collected to analyze the change in the relative expression of PtCyP1, Data are presented as the means ± standard deviation (SD) from three biological replicates. Different letters indicate significant differences (*P <* 0.05). (**B**) Relative expression levels of PtCyP1 gene in *P.thunbergii* infected with dsRNA-treated nematodes. At 12 h, the *P. thunbergii* stem samples were collected to analyze the change in the relative expression of PtCyP1, Data are presented as the means ± standard deviation (SD) from three biological replicates. Different letters indicate significant differences (*P <* 0.05). (**C**) Immunoblot analysis of purified BxML1 and GFP protein (This picture has been cropped. For full length original blot, see Additional file 7). (**D**) Relative expression levels of PtCyP1 gene in *P.thunbergii* infected with purified BxML1 protein and PWN. At 12 h, the *P. thunbergii* stem samples were collected to analyze the change in the relative expression of PtCyP1, Data are presented as the means ± standard deviation (SD) from three biological replicates. Different letters indicate significant differences (*P <* 0.05)

To explore whether PtCyP1 reacts to nematode infection in pine, the expression levels of PtCyP1 were measured in nematode-infected at 12 h. Our results demonstrated that PtCyP1 expression was upregulated 4-fold after nematode inoculation (Fig. 7A). We therefore speculated that PtCyP1 may be involved in the defence response of pine to *B. xylophilus* infection.

To further explore the effect of BxML1 on PtCyP1, the expression of PtCyP1 was measured in *P. thunbergii* infected with dsBxML1-treated PWNs. RT-qPCR analysis showed that the expression of PtCyP1 was reduced in pine infected with dsBxML1-treated PWNs compared with that in pine infected with WT-treated and dsGFP-treated PWNs (Fig. 7)B. The yeast expression system is a powerful tool for the production of recombinant eukaryotic proteins. We cloned, expressed and purified the BxML1 proteins through a yeast expression system. The BxML1 and GFP proteins were assessed by sodium dodecyl sulfate-polyacrylamide gel electrophoresis (SDS-PAGE) and western blot analysis to confirm successful purification (Fig. 7C, S3). We injected purified BxML1 protein into the stem of *P. thunbergii* and performed PWN inoculation 2 h later. Purified GFP was used as a negative control. The results showed that compared with the negative control, the expression of PtCyP1 decreased in pine trees inoculated with purified BxML1 protein (Fig. 7D). The above results showed that the BxML1 protein may inhibit the expression of PtCyP1 in *P. thunbergii*.

## Discussion

Effectors are thought to play an important role in the pathogen infection process [31]. Our previous studies demonstrated that several *B. xylophilus* effectors could suppress cell death and plant defences [23, 24]. We screened a potential effector, BxML1, containing an ML domain, from the transcripts of *P. thunbergii* infected by *B. xylophilus*. ML domain-containing proteins are recognized as immune-related molecules. Previous studies have shown that all ML proteins possess a putative N-terminal SP sequence and two pairs of conserved cysteine residues [28]. The predicted BxML1 protein had typical structural features of ML proteins, and this was confirmed by sequence alignment, functional domain and 3D structure analyses. Little is known about the molecular features of ML proteins in nematodes, especially in the process of interaction with host plants.

The functionality of BxML1 supresses PTI, as experimentally demonstrated by transiently expressing BxML1 and BxCDP1, which are described as molecular patterns in *B. xylophilus* [19]. This suggests that BxML1 is a potential effector of *B. xylophilus*. Subcellular localization is important for the functional regulation of effector proteins [32]. Our results revealed that BxML1 exhibited cytoplasmic and nuclear distribution in *N. benthamiana* leaf cells. Most effectors have been shown to be targeted to the nucleus, cytosol, plasma membrane, or endoplasmic reticulum. Some effectors require specific cellular localization to exert virulence [24]. To identify the subcellular localization that leads to activation of BxML1 virulence, we conducted further studies using NLSs/NESs. However, our results indicated that although BxML1 exhibited cytoplasmic and nuclear distribution in *N. benthamiana* leaf cells, it did not depend on a specific subcellular localization to suppress BxCDP1-induced cell death. We speculate that BxML1 may have a variety of strategies to suppress the PTI response.

Moreover, the expression level of BxML1 was highly upregulated after 12 h of pine infection, suggesting that BxML1 may play an important role in infection in the phytophagous parasitic stage. Most effectors are secretory proteins (SP) [33]. When PPNs invade plants, the effector SPs are transported to the endoplasmic reticulum (ER) for secretion. In situ hybridization showed that BxML1 was expressed in the dorsal glands and intestine of *B. xylophilus*. The dorsal glands are the main source of secretory proteins. Recent studies have suggested that most PPN effectors are secreted by dorsal glands [34]. The intestine is the less important secretory source of effectors in the PPN. However, it is a large organ that encompasses almost the entire body cavity, which strongly suggests a significant role for BxML1 in *B. xylophilus*. PPN effectors originate from diverse organs, such as oesophageal glands, amphids or cuticles [16, 35, 36], and *B. xylophilus* exhibits the same characteristics. For instance, BxSapB2 is expressed in the oesophageal glands and amphids of *B. xylophilus* [21]. Bx-FAR-1 is expressed in the median bulb and seminal vesicle of male nematodes and the intestine of female nematodes and juveniles [23]. These effectors were upregulated in the early interaction between *B. xylophilus* and pine. Our present study further shows the diversity of effector expression patterns. Therefore, we inferred that BxML1 is a secretory protein, a new effector of *B. xylophilus*, and participates in the interaction between *B. xylophilus* and pine.

Feeding and reproduction are important conditions for the virulence of *B. xylophilus* [37]. Our results demonstrated that RNAi-mediated BxML1 expression reduced the suppression of *B. xylophilus* feeding and fecundity on *B. cinerea*. Moreover, the appearance of symptoms in *P. thunbergii* was delayed after infection with BxML1-silenced *B. xylophilus*. PRs are an important class of inducible plant disease defence proteins, and they function as key players in an immune surveillance mechanism that protects plants primarily against invasion by microorganisms [38–40]. Inoculation of *B. xylophilus* in which the effector is silenced will lead to differences in the expression of PR genes in pine [23]. Our study showed that silencing of BxML1 could upregulate the expression of multiple pine PR genes. Notably, the expression of the PR1 gene was much higher in pine inoculated with BxML1-silenced nematodes than in the control. PR1 was first found in tobacco [41], is regarded as a marker gene involved in plant systematic acquired resistance and can improve the ability of plants to tolerate multiple biotic and abiotic stresses [42, 43]. Some PR1 proteins have been proven to have activity against phytopathogens such as bacteria, fungi, and oomycetes [44–46]. PPN infection also causes upregulation of PR1 expression. For example, the expression of the PR1 gene in tomato was significantly upregulated after infection with potato cyst nematode [47]. Therefore, we speculated that BxML1 attenuates multiple defences of pine trees and promotes the process of PWN infection. Collectively, our research revealed that BxML1 plays a vital role in the feeding, reproduction and virulence of *B. xylophilus*.

Using a Y2H screen, we identified a cyclophilin protein (PtCyP1) as a potential target of BxML1. The CyP protein family is widely present in humans, animals, plants, fungi, and bacteria and is involved in many cellular biological functions, including protein trafficking and maturation [48], receptor signalling [49], immunomodulatory activity [50] and apoptosis [51]. Response to environmental stresses such as drought, salinity stress and low temperature is a significant characteristic of CyP [52–54]. In addition, the expression of CyP is induced by pathogens. For instance, fungal infection increased StCyP levels in potato tubers [55]. Nevertheless, CCyP, isolated from Chinese cabbage, exhibited antifungal activity [56]. These examples indicate that CyP may have broad functions in plant development and stress responses. Our results showed the upregulation of PtCyP1 expression during *B. xylophilus* infection. This indicates that PtCyP1 may be involved in the defence response during *B. xylophilus* infection. Silencing of BxML1 led to upregulation of PtCyP1 expression. However, the addition of pure BxML1 protein had the opposite result. The above results suggest that the interaction between BxML1 and PtCyP1 affected the expression of PtCyP1 and then affected the ability of pine to resist *B. xylophilus* infection.

## Conclusions

Here, we determined that the PWN effector BxML1 contains an ML domain, which can suppress PTI. In addition, BxML1 plays an important role in the feeding, reproduction and virulence of *B. xylophilus*. We confirmed through Y2H screening that BxML1 can target and regulate the PtCyP1 protein in pine. Based on our study, we analysis interaction between *B. xylophilus* and host. We find BxML1 plays a critical role in the virulence of *B. xylophilus.* Thus, we hope contribute to initially develop new strategies to control the PWN from aspects of weakening colonization and virulence of *B. xylophilus* in host plant.

## Methods

### Nematode culture and plant material

The *B. xylophilus* AMA3 population was originally isolated from diseased *Pinus massoniana* in Anhui Province, China. Nematodes were cultured on PDA plates covered with *Botrytis cinerea* mycelia at 25 °C for 7 days. *B. xylophilus* was collected on a modified Baermann funnel by a previously described technique.

Seeds of *Nicotiana benthamiana* provided by Prof. Yuanchao Wang (Nanjing Agricultural University). *Nicotiana benthamiana* was grown in the greenhouse of Nanjing Forestry University (NJFU) at 25 °C with a cycle of 16 h of high light intensity and 8 h of darkness. *Pinus thunbergii* seedlings (3 years old) obtained from the experimental field of Nanjing Forestry University (Jurong Yaolingkou Forest Farm, Jiangsu, China), then planted in the greenhouse of Nanjing Forestry University. All plant materials used in this study were collected in compliance with local regulations. All the plants used in this research are planted in Nanjing Forestry University and none of the are wild. Voucher specimen of *P. thunbergii* deposited in NJFU (voucher number pt-20190403).

### BxML1 gene cloning and real-time RT-qPCR analyses

Total RNA was extracted from *B. xylophilus* using TRIzol Reagent (Invitrogen, Carlsbad, California, USA) according to the manufacturer’s instructions. RNA concentration and purity were analysed by a NanoDrop® ND-2000 spectrophotometer (Thermo Fisher Scientific). cDNA was synthesized using 1 μg of total RNA by HiScript II Q RT SuperMix for qPCR according to the manufacturer’s instructions (Vazyme, Nanjing, China).

Based on *B. xylophilus* transcriptomic data [29], the coding sequence of BxML1 was amplified from *B. xylophilus* cDNA using specific primers. The primers for qPCR were designed on the NCBI website. (All the primers are listed in Table S1) Quantitative PCR (qPCR) was carried out as described previously [57].

### Alignment and protein modelling

The protein sequence of BxML1 of *B. xylophilus* was deduced from the nucleotide sequence of the cloned gene. The protein sequences for additional species used in the alignments were retrieved from GenBank (www.ncbi.nlm.nih.gov/genbank/) or Wormbase (wormbase.org). The prediction of transmembrane and conserved domains was performed using TMHMM (http://www.cbs.dtu.dk/services/TMHMM-2.0/) and NCBI CD-Search (https://www.ncbi.nlm.nih.gov/Structure/cdd/wrpsb.cgi), respectively. Putative SP analysis was performed using SignalP 4.0 (http://www.cbs.dtu.dk/services/SignalP/). Multiple sequence alignments were performed with DNAMAN software (All the sequence are listed in Table S2).

### Transient expression of BxML1 in plants

The BxML1 gene was cloned using cDNA from *B. xylophilus*. The amplified fragments were ligated into pBINRFP (a plasmid containing RFP) and PVX (pGR107) using the appropriate restriction enzymes and a Clone Express II One Step Cloning Kit (Vazyme). BxML1 mutants were amplified using combinations of primers. Individual colonies for each construct were tested for inserts by PCR, and selected clones were verified by sequencing.

The confirmed constructs were introduced into *Agrobacterium tumefaciens* strain GV3101 by electroporation. The *A. tumefaciens* cells were resuspended in wash buffer (10 mM MgCl_2_, 10 mM MES, 100 μM acetosyringone; pH 5.6) and diluted to a final optical density (OD) at 600 nm of 0.4–0.6. The identical infiltration site was then challenged with *A. tumefaciens* cells carrying the BxCDP1 gene at 16 h after initial inoculation. Simultaneously, the BxCDP1 gene was expressed alone as a control. Each experiment was performed on six leaves from three individual plants and repeated at least three times.

### Electrolyte leakage assay

Electrolyte leakage in the inoculated *N. benthamiana* leaves was measured as described previously [19]. Electrolyte leak assays is performed by Five Easy Plus FE28(METTLER TOLEDO). All assays were repeated three times.

### Total protein extraction and western blot analysis

Proteins were prepared from the infiltrated tissues collected 36 h after agroinfiltration. The tissues were ground in liquid nitrogen, and the total proteins were extracted using lysis buffer (10 mM Tris-HCl [pH 7.5], 150 mM NaCl, 0.5% NP-40), 1 mM phenylmethylsulfonyl fluoride [PMSF], 0.5 mM ethylenediaminetetraacetic acid [EDTA], and a protease inhibitor cocktail; Sigma-Aldrich) by mixing 1 g of leaf tissue with 2 ml of lysis buffer. The samples were centrifuged at 4 °C and 12,000×g for 15 min, and the supernatant was transferred to a new tube.

Nitrocellulose membranes were blocked with 50 mM Tris-HCl (pH 7.5) containing 150 mM NaCl, 0.1% Tween 20, and 2% milk powder. The membranes were incubated with the primary antibodies for 3 h at 1:2000 (HA-tagged antibodies; Sigma-Aldrich), followed by incubation with the secondary antibodies for 1 h at 1:10,000. Detection was performed by chemiluminescence using an ECL kit according to the manufacturer’s instructions (Pierce).

### In situ hybridization

mRNA in situ hybridization using digoxigenin-labelled probes was performed to determine the spatial expression patterns of BxML1 in *B. xylophilus*. A 501-bp gene was used to generate/synthesize probes. Antisense and sense DIG-labelled probes were prepared separately by unidirectional PCR with these templates. In situ hybridization was performed as described previously [58] using a DIG High Prime RNA Labeling and Detection Starter Kit I (Roche Diagnostics). Finally, the samples were observed under an Axio Image M2 microscope (Zeiss).

### Subcellular localization

BxML1 and its mutants cloned into pBinRFP were transformed into 4-week-old *N. benthamiana* leaves by agroinfiltration. Plants were grown in a growth chamber at 25 °C under 16 h:8 h light:dark conditions for 2 days. To observe fluorescence, patches of *N. benthamiana* leaves were analysed using a confocal laser scanning microscope (Zeiss).

### Synthesis of dsRNA

The dsRNA for BxML1 was obtained using the MEGAscript® T7 High Yield Transcription Kit (Thermo Fisher Scientific) according to the manufacturer’s instructions. The quantity and quality of the synthesized dsRNA were determined by a NanoDrop® ND-2000 spectrophotometer (Thermo Fisher Scientific, USA) and gel electrophoresis, respectively. Subsequently, approximately 10,000 *B. xylophilus* (mixture of juveniles and adults) individuals were soaked in 50 μL of BxML1 dsRNA and GFP dsRNA (both at 800 ng/μL) for 48 h in a rotator at 20 °C in the dark. To analyse the effect of RNAi, approximately 2000 nematodes from each treatment were used to extract RNA and synthesize cDNA as described previously. The cDNA was used for qRT-PCR to evaluate the effect of RNAi with the appropriate primers.

### Reproduction test

Adult male and female J4 nematodes were obtained and soaked in buffer with BxML1 dsRNA and GFP dsRNA for 24 h. To investigate the effect of BxML1 on progeny production, 40 female nematodes and 40 male nematodes were inoculated on *B. cinerea*-colonized PDA plates. After inoculation, the PDA plates were cultured in the dark at 25 °C for 6 days. Subsequently, the Baermann funnel method was used to collect all nematodes from the PDA plates. The number of nematodes was counted with an optical microscope (Leica DM500, Leica Microsystems, Heerbrugg, Switzerland).

### PWN inoculation and sampling

A sterile blade was used to cut a small wound deep into the xylem on two-year-old pine stems, and a sterile cotton ball was inserted. The incision and cotton ball were then covered with a funnel-shaped parafilm. Approximately 10,000 nematodes were soaked in BxML1 dsRNA, GFP dsRNA and non-dsRNA solution. Pine stem segments ~ 2 cm in length at 1 cm below the inoculation points were cut and frozen in liquid nitrogen for RNA extraction.

### Y2H and CoIP assays

For the Y2H screens, BxML1 (without an SP) was cloned into the pGBKT7 bait vector. The constructs were verified by sequencing and used to transform the yeast strain Y2H Gold. These baits were used to screen a randomly primed cDNA library constructed from *B. xylophilus*-infected pine. Subsequently, BxML1 was cloned into the pGBKT7 vector, and potential interaction partners were cloned into the pGADT7 vector to perform a Y2H assay.

For the coimmunoprecipitation (CoIP) assay, the BxML1 and PtCyP1 sequences were inserted into the PVX vector and pBINGFP vector and introduced into *A. tumefaciens* GV3101, which was then used to transform *Nicotiana tabacum* leaves. Cell suspensions (PVX-BxML1 and pBINGFP-PtCyP1) and mixed bacterial solutions (PVX-BxML1 and pBINGFP-PtPtCyP1) were infiltrated into *N. benthamiana*. At 48 h after infiltration, total protein was extracted from *N. tabacum* leaves. CoIP was performed as described previously [59].

### Expression of the recombinant BxML1 protein

BxML1 (without an SP) and GFP were cloned into pPICZαA, which encodes a 6 × His tag. The ligated vectors were transformed into competent *Escherichia coli* DH5α cells. Individual colonies from the construct were tested by PCR for insertions, and the selected clones were verified by sequencing. The recombinant vectors were transformed into *Pichia pastoris* KM71H (Muts) (Invitrogen) by electroporation. Positive clones were grown in yeast extract-peptone-dextrose (YPD) medium containing 100 μg/mL zeocin at 30 °C for 3 days. Buffered glycerol complex medium (BMGY) and buffered methanol complex medium (BMMY) were used for protein expression, which was verified by SDS-PAGE. Purification of the recombinant protein BxML1-6 × His and GFP from the culture supernatant was performed by affinity chromatography using Ni-NTA Superflow resin (Qiagen).

## Supplementary Information

**Additional file 1.**

**Additional file 2.**

**Additional file 3.**

**Additional file 4.**

**Additional file 5.**

**Additional file 6.**

**Additional file 7.**

**Additional file 8.**

## Data Availability

All data generated or analysed during this study are included in this published article. The RNA-seq data in this study was available through the NCBI under accession number PRJEB40022.

## Abbreviations

PWN: Pine wood nematode
PWD: Pine wilt disease
PAMPs: Pathogen-associated molecular patterns
PRRS: Pattern recognition receptors
PTI: PAMP-triggered immunity
ETI: Effector-triggered immunity
SP: Secretory proteins
ER: Endoplasmic reticulum
ISH: In situ hybridization
PRs: Pathogenesis-related proteins
Y2H: Yeast two-hybrid
