# Supplementary material for: The Bursaphelenchus xylophilus effector BxML1 targets the cyclophilin protein (CyP) to promote parasitism and virulence in pine
Source: BMC Plant Biol. 2022 Apr 27;22:216. doi: 10.1186/s12870-022-03567-z (PMC9044635; doi:10.1186/s12870-022-03567-z)
Supplement: Supplementary file 1 — Additional file 1. [file 12870_2022_3567_MOESM1_ESM.docx]

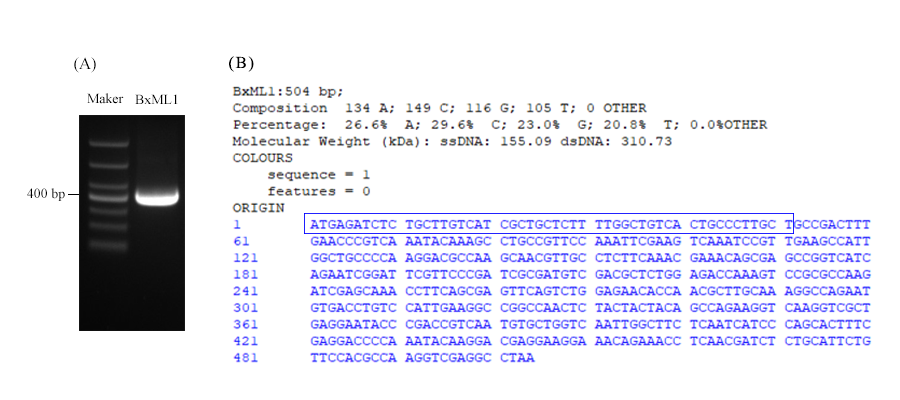


**Figure S1:** Cloning and sequencing of BxML1, which upregulated in the infection stage. (A) Agarose gel electrophoresis of BxML1 gene. (B) Sequences of BxML1, the blue box indicates the predicted SP of BxML1.
