# Supplementary material for: The Bursaphelenchus xylophilus effector BxML1 targets the cyclophilin protein (CyP) to promote parasitism and virulence in pine
Source: BMC Plant Biol. 2022 Apr 27;22:216. doi: 10.1186/s12870-022-03567-z (PMC9044635; doi:10.1186/s12870-022-03567-z)
Supplement: Supplementary file 2 — Additional file 2. [file 12870_2022_3567_MOESM2_ESM.docx]

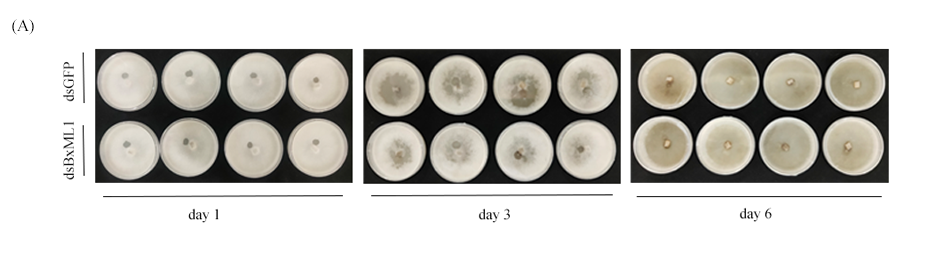


Figure S2: The effect of BxML1 silencing on the feeding rate of *Bursaphelenchus xylophilus*. Difference in feeding of dsBxML1 and dsGFP cultured on Botrytis cinerea.
