# Supplementary figures and images for: The Bursaphelenchus xylophilus effector BxML1 targets the cyclophilin protein (CyP) to promote parasitism and virulence in pine
Source: BMC Plant Biol. 2022 Apr 27;22:216. doi: 10.1186/s12870-022-03567-z (PMC9044635; doi:10.1186/s12870-022-03567-z)

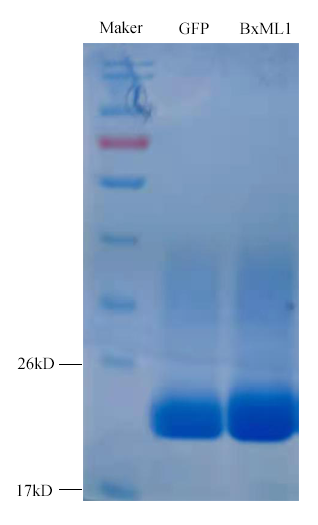


**Figure S3:** SDS gel of the purified BxML1 and GFP protein.

Supplement: Supplementary file 3 — Additional file 3. [file 12870_2022_3567_MOESM3_ESM.docx]
