# Supplementary material for: The Bursaphelenchus xylophilus effector BxML1 targets the cyclophilin protein (CyP) to promote parasitism and virulence in pine
Source: BMC Plant Biol. 2022 Apr 27;22:216. doi: 10.1186/s12870-022-03567-z (PMC9044635; doi:10.1186/s12870-022-03567-z)
Supplement: Supplementary file 4 — Additional file 4. [file 12870_2022_3567_MOESM4_ESM.docx]

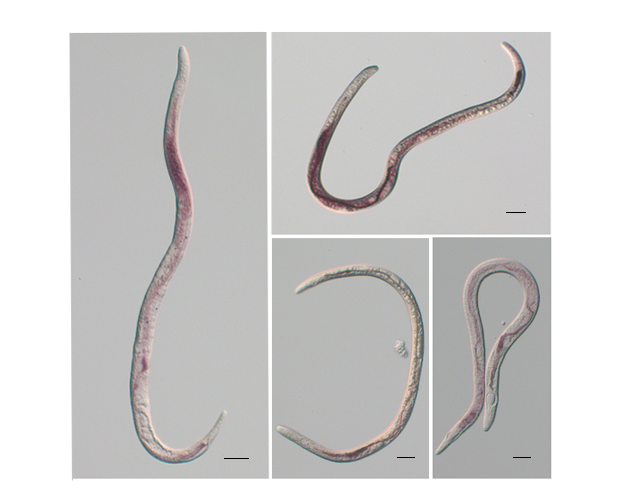


**Figure S4:** Localization of BxML1 in the dorsal glands (DG) and intestine of PWN by in situ hybridization. Scale bars = 20 µm.
