# Supplementary material for: The Bursaphelenchus xylophilus effector BxML1 targets the cyclophilin protein (CyP) to promote parasitism and virulence in pine
Source: BMC Plant Biol. 2022 Apr 27;22:216. doi: 10.1186/s12870-022-03567-z (PMC9044635; doi:10.1186/s12870-022-03567-z)
Supplement: Supplementary file 5 — Additional file 5. [file 12870_2022_3567_MOESM5_ESM.docx]

Table S1. List of gene specific primers used in this work

| Primers | Sequence |
| --- | --- |
| pGR107-BxML1-F | CTAGCATCGATTCCCGGGGCCGACTTTGAACCCGTC |
| pGR107-BxML1sp-F | CTAGCATCGATTCCCGGGATGAGATCTCTGCTTGTCA |
| pGR107-BxML1-R | CTCTAGAGGATCCCCGGGGGCCTCGACCTTGGCGTG |
| BxML1-F for qPCR | ATACAAAGCCTGCCGTTCCA |
| BxML1-R for qPCR | GGCTCGCTGTTTCGTTTGAA |
| Actin-F | GCAACACGGAGTTCGTTGTA |
| Actin-R | GTATCGTCACCAACTGGGAT |
| ISH-BxML1-F | TAATACGACTCACTATAGGGGGCCTCGACCTTGGCGTG |
| ISH-BxML1-R | ATGGCCGACTTTGAACCCGTC |
| dsGFP-F | TAATACGACTCACTATAGGGATGAGTAAAGGAGAAGAACTTTTC |
| dsGFP-R | TAATACGACTCACTATAGGGTTTGTATAGTTCATCCATGCCAT |
| dsBxML1-F | TAATACGACTCACTATAGGGATGGCCGACTTTGAACCCGTC |
| dsBxML1-R | TAATACGACTCACTATAGGGGGCCTCGACCTTGGCGTG |
| pGBKT7-BxML1-F | ATGGAGGCCGAATTCCCGGGATGGCCGACTTTGAACCCGTC |
| pGBKT7-BxML1-R | CAGGTCGACGGATCCCCGGGGGCCTCGACCTTGGCGTG |
| pGADT7-PtCyp1-F | CCCACCCGGGTGGAATTCGAGCTGACCGCAATCAGCAATAAC |
| pGADT7-PtCyp1-R | ATGGAGGCCAGTGAATTCATGCCGAACCCTAAGGTTTA |
| PtCyp1-F for qPCR | GCAGAGAACTTCCGAGCACT |
| PtCyp1-R for qPCR | GTATTCTTCCCGGCGTTTGC |
| PtEF1α-F | GGGAAGCCACCCAAAGTTTT |
| PtEF1α-R | TACATGGGAAGACGCCGAAT |
| PPICZαA- BxML1-F | CGGGGTACCATGAGATCTCTGCTTGTCATC |
| PPICZαA- BxML1-R | CTAGTCTAGAGGCCTCGACCTTGGCGTG |

**Table S1:** List of gene specific primers used in this work
