# Supplementary material for: The Bursaphelenchus xylophilus effector BxML1 targets the cyclophilin protein (CyP) to promote parasitism and virulence in pine
Source: BMC Plant Biol. 2022 Apr 27;22:216. doi: 10.1186/s12870-022-03567-z (PMC9044635; doi:10.1186/s12870-022-03567-z)
Supplement: Supplementary file 6 — Additional file 6. [file 12870_2022_3567_MOESM6_ESM.docx]

Table S2. The Sequence ID and gene information of the secreted homologs of BxML1 in the sequence alignment

| Organism | Sequence ID | Amino acid sequence length |
| --- | --- | --- |
| *Bursaphelenchus okinawaensis* | CAD5207538.1 | 166 |
| *Brugia malayi* | XP 001898567.2 | 165 |
| *Haemonchus contortus* | CDJ85715.1 230 | 230 |
| *Caenorhabditis elegans* | NP 001379800.1 | 154 |
| *Chilo suppressalis* | RVE48176.1 | 146 |
| *Danaus plexippus* | XP 032518293.1 | 145 |

**Table S2:** The Sequence ID and gene information of the secreted homologs of BxML1 in the sequence alignment
