# Supplementary material for: The Bursaphelenchus xylophilus effector BxML1 targets the cyclophilin protein (CyP) to promote parasitism and virulence in pine
Source: BMC Plant Biol. 2022 Apr 27;22:216. doi: 10.1186/s12870-022-03567-z (PMC9044635; doi:10.1186/s12870-022-03567-z)
Supplement: Supplementary file 7 — Additional file 7. [file 12870_2022_3567_MOESM7_ESM.docx]

**
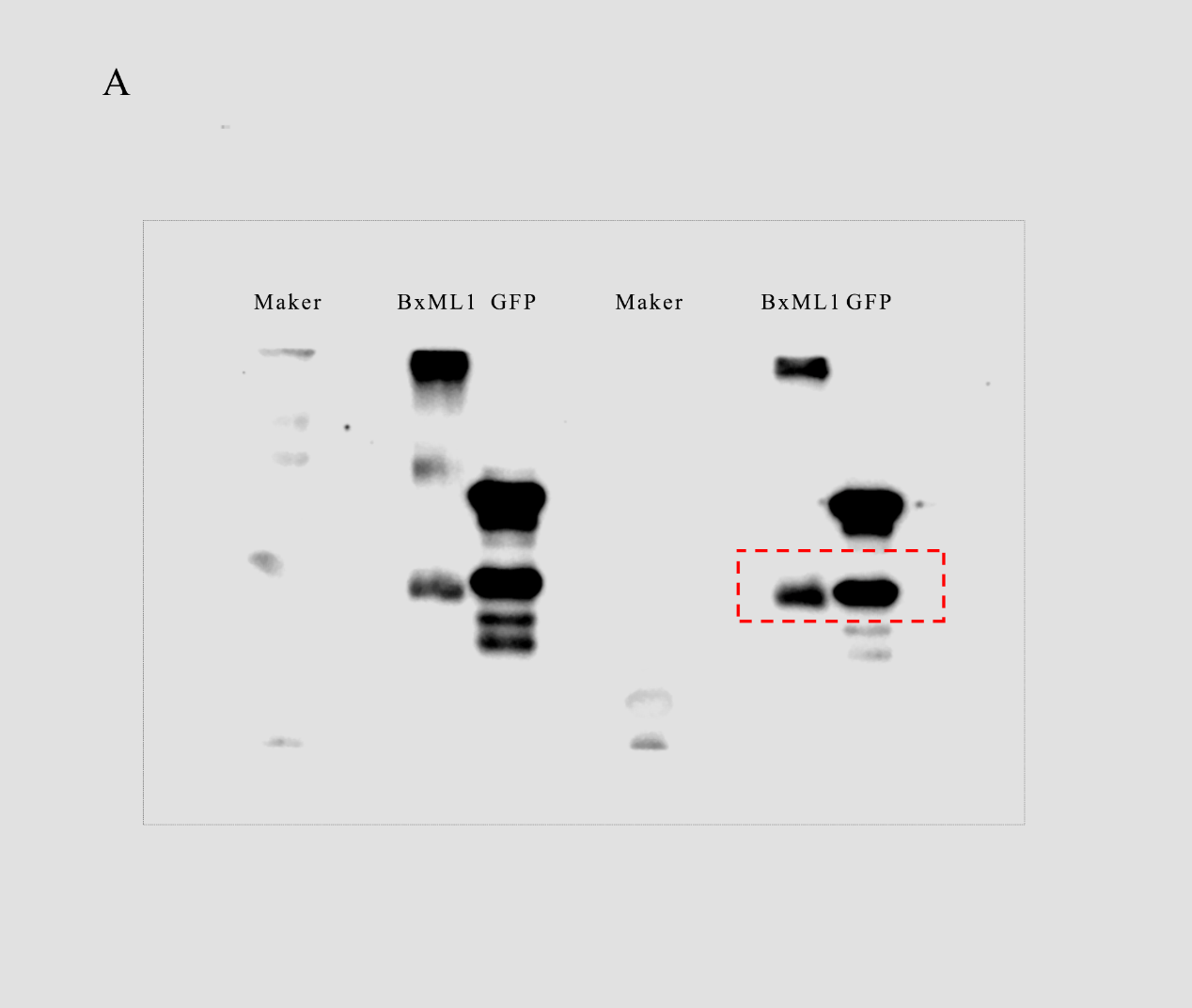
**

**
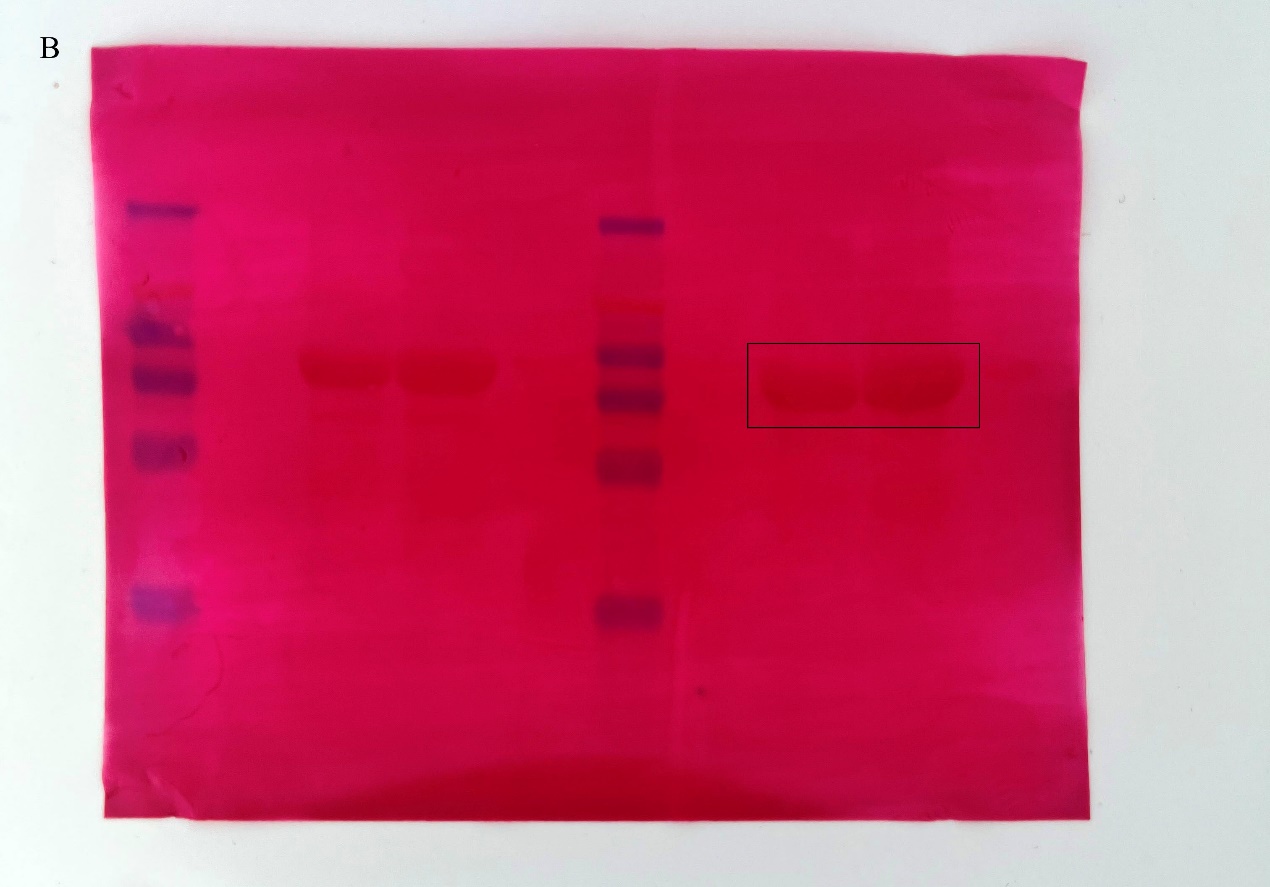
**

**
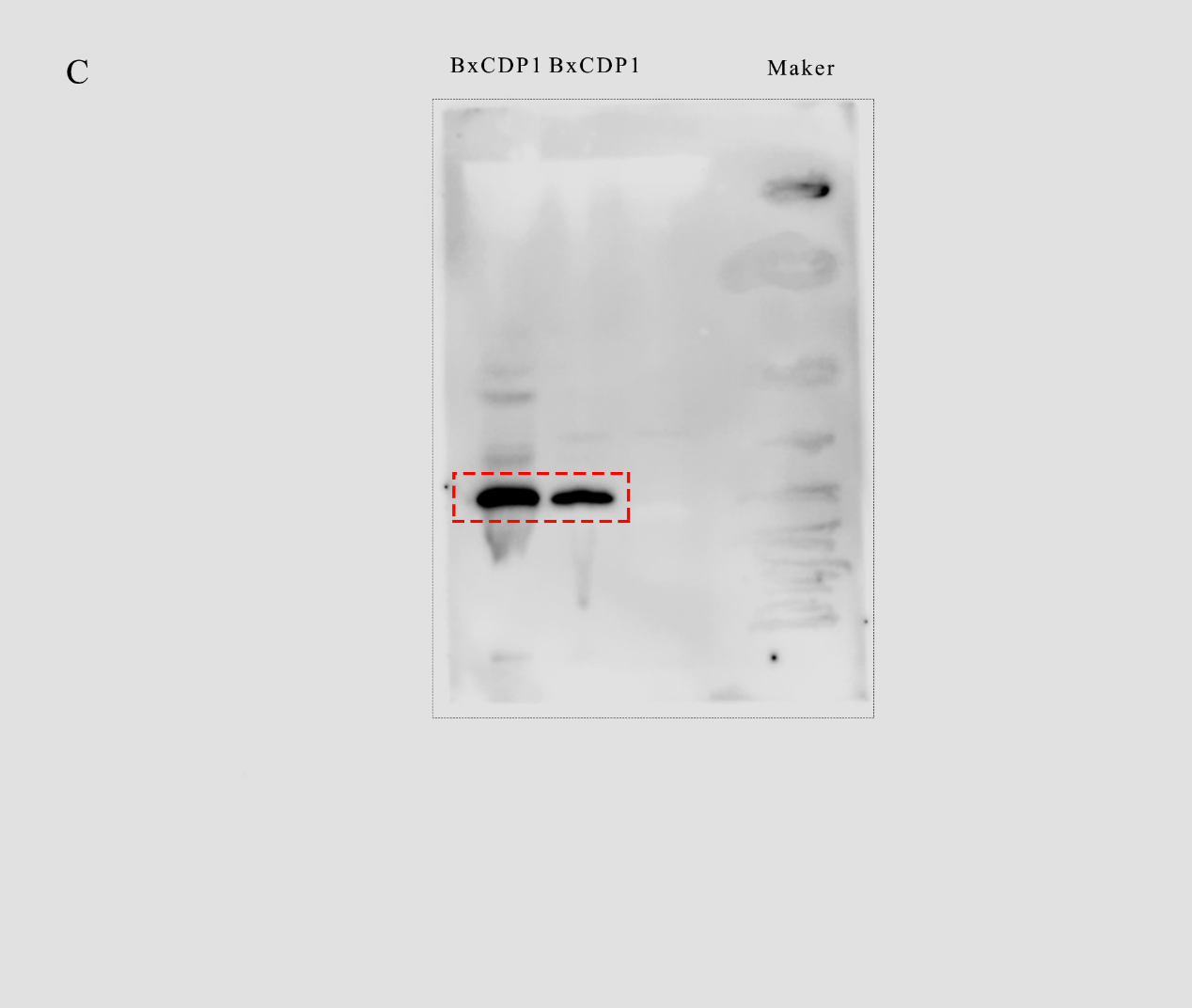
**

**
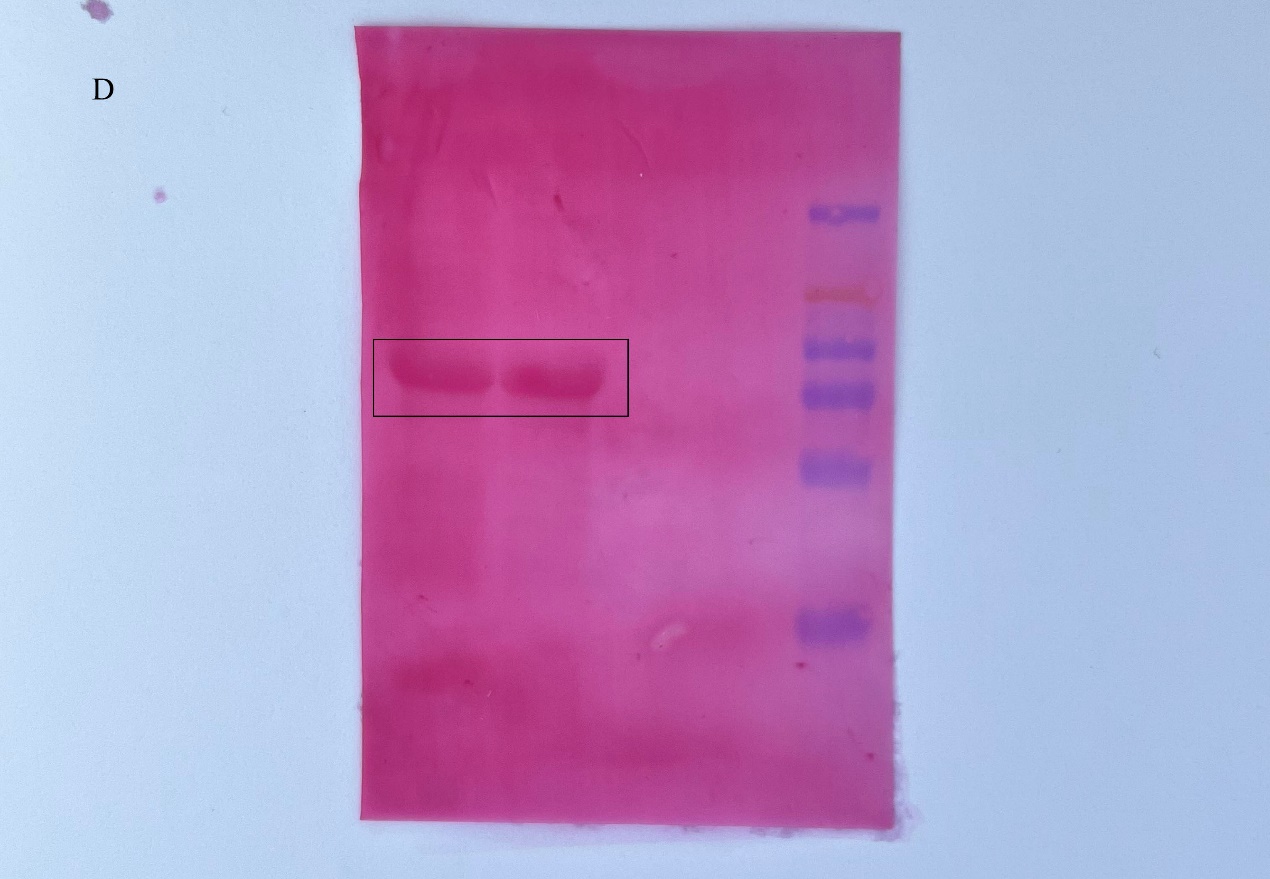
**

**Figure A-D: Supplement of** Figure 2B. Figure A: Immunoblot analysis of BxML1 and GFP proteins from *N. benthamiana* leaves transiently expressing target proteins (The black box indicates the boundary between the gels and rad box indicates the regions used in the main figures). Figure B: Ponceau S of Figure A (The black box indicates the regions used in the main figures). Figure C: Immunoblot analysis of BxCDP1 proteins from *N. benthamiana* leaves transiently expressing target proteins (The black box indicates the boundary between the gels and rad box indicates the regions used in the main figures). Figure D: Ponceau S of Figure C (The black box indicates the regions used in the main figures).


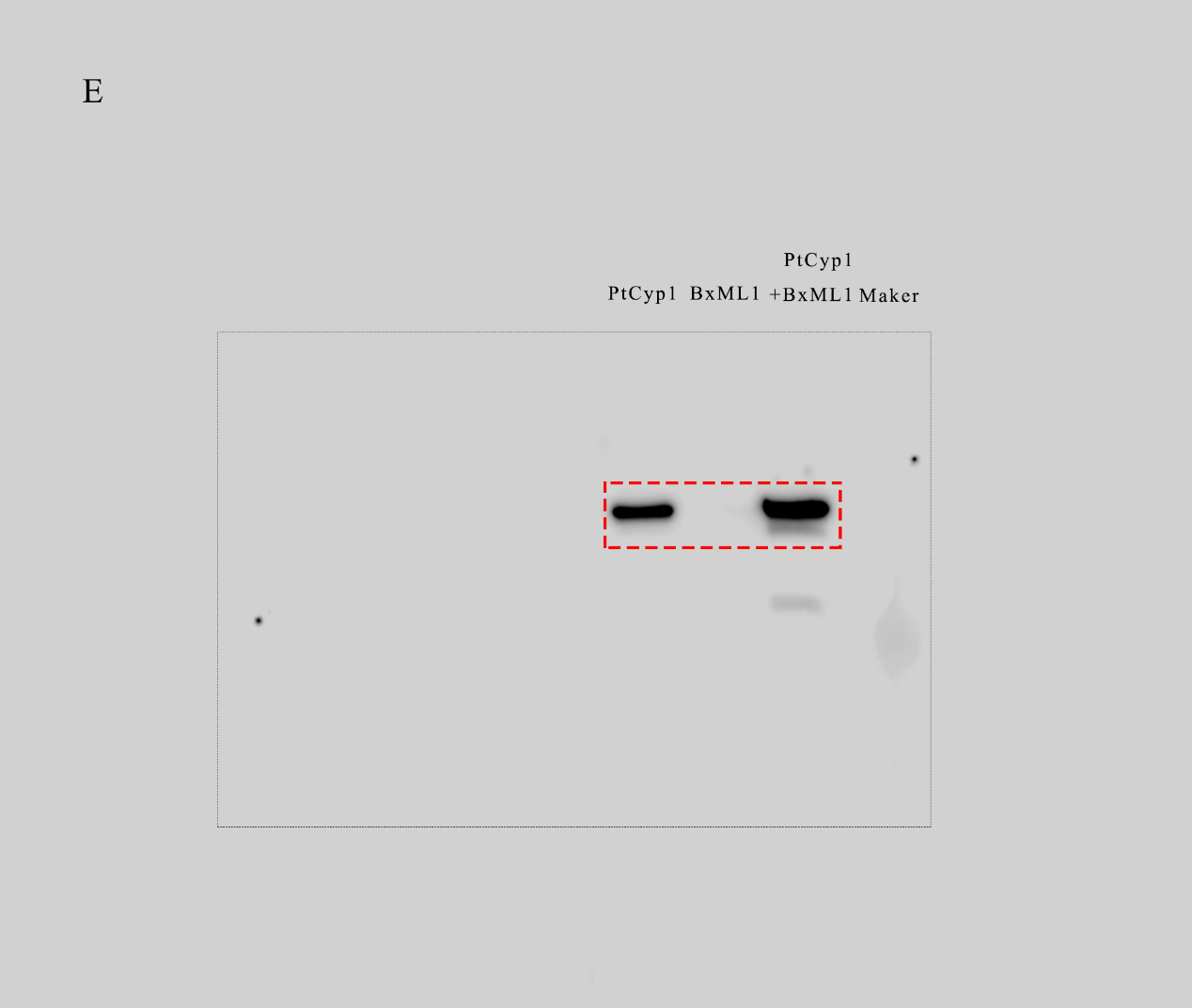


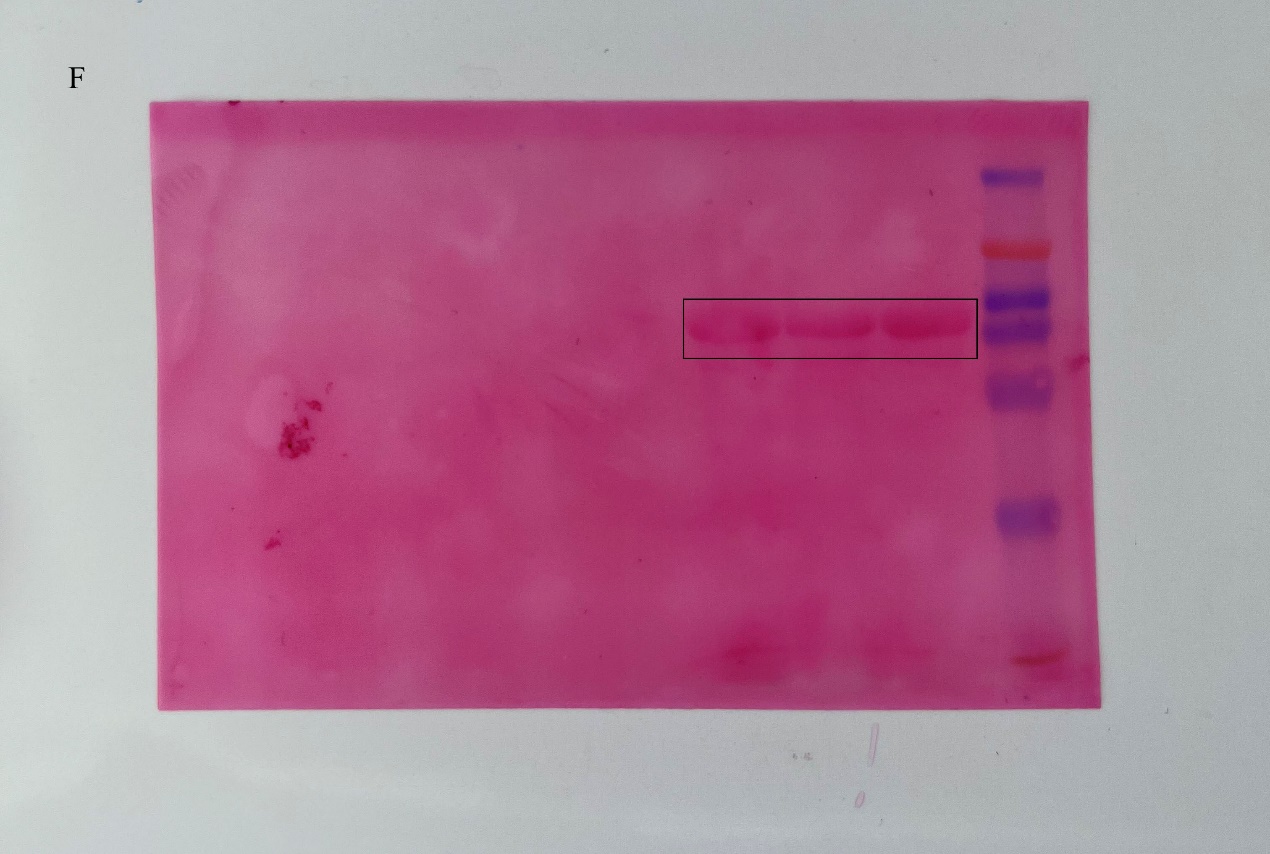


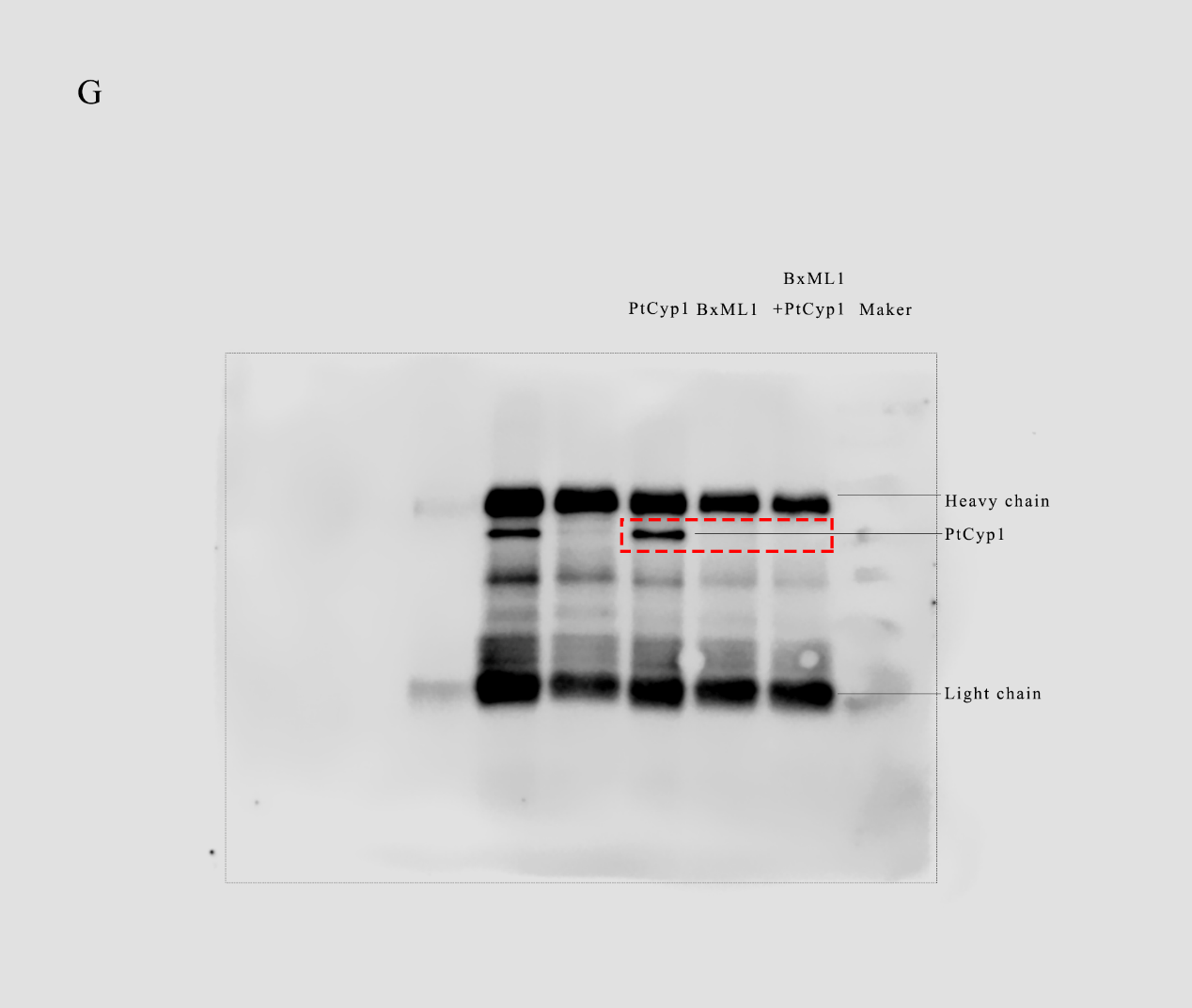


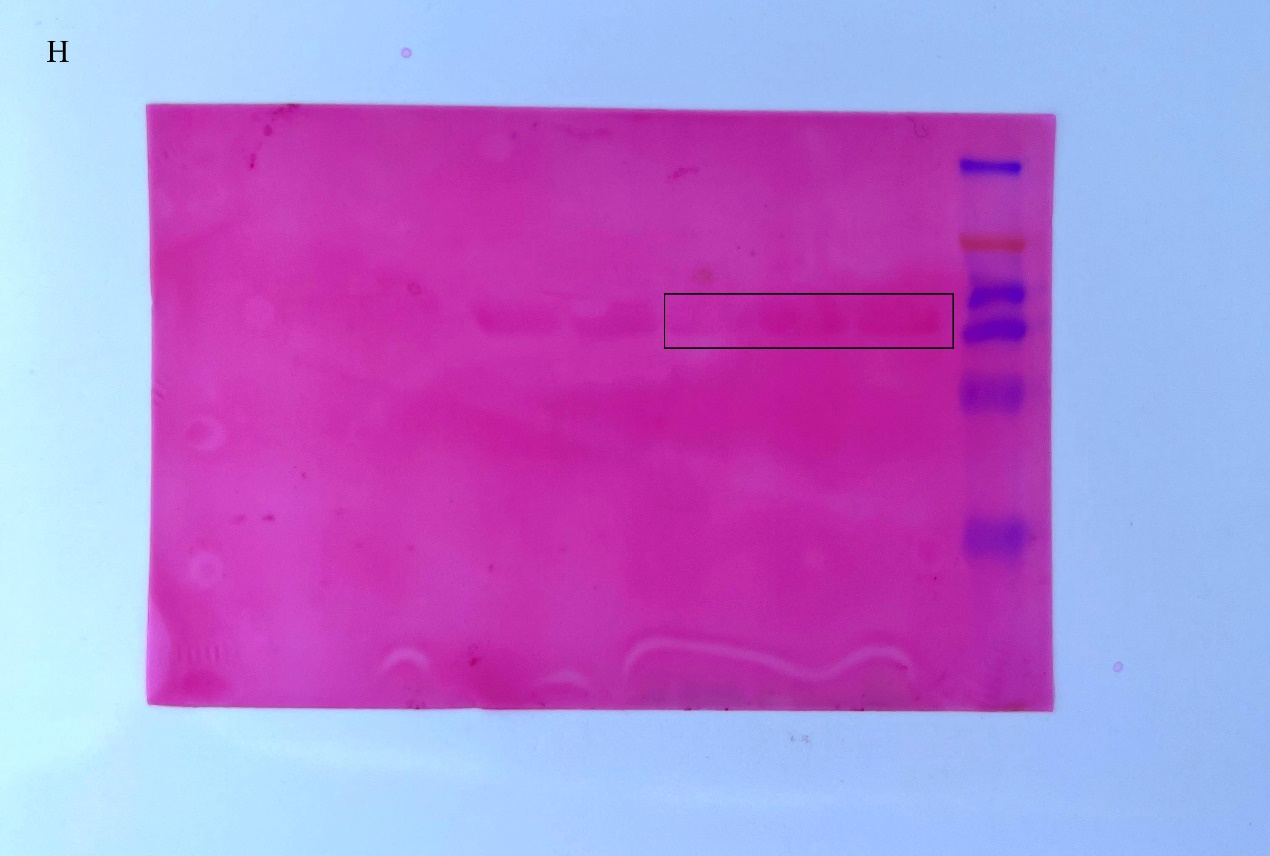


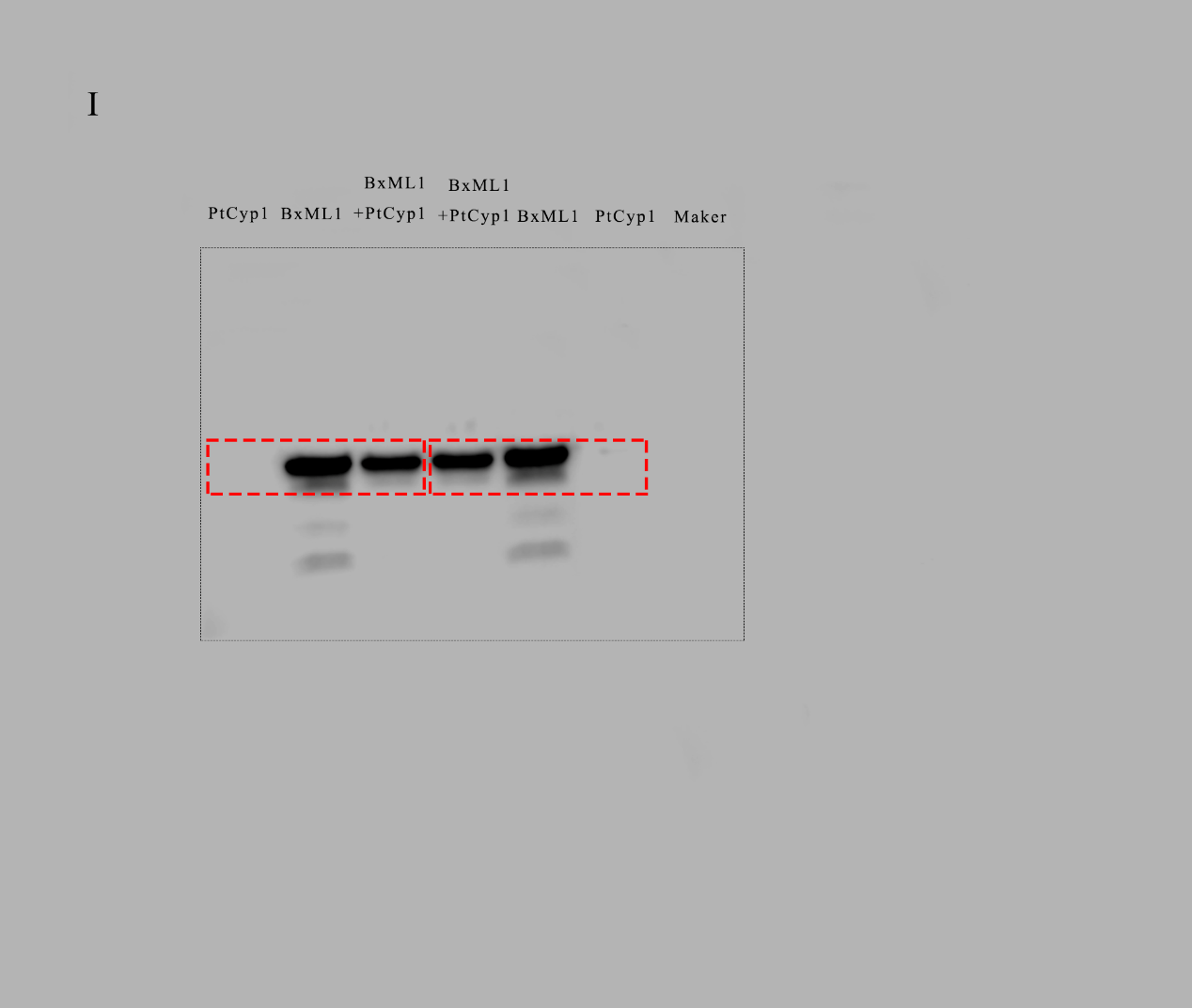


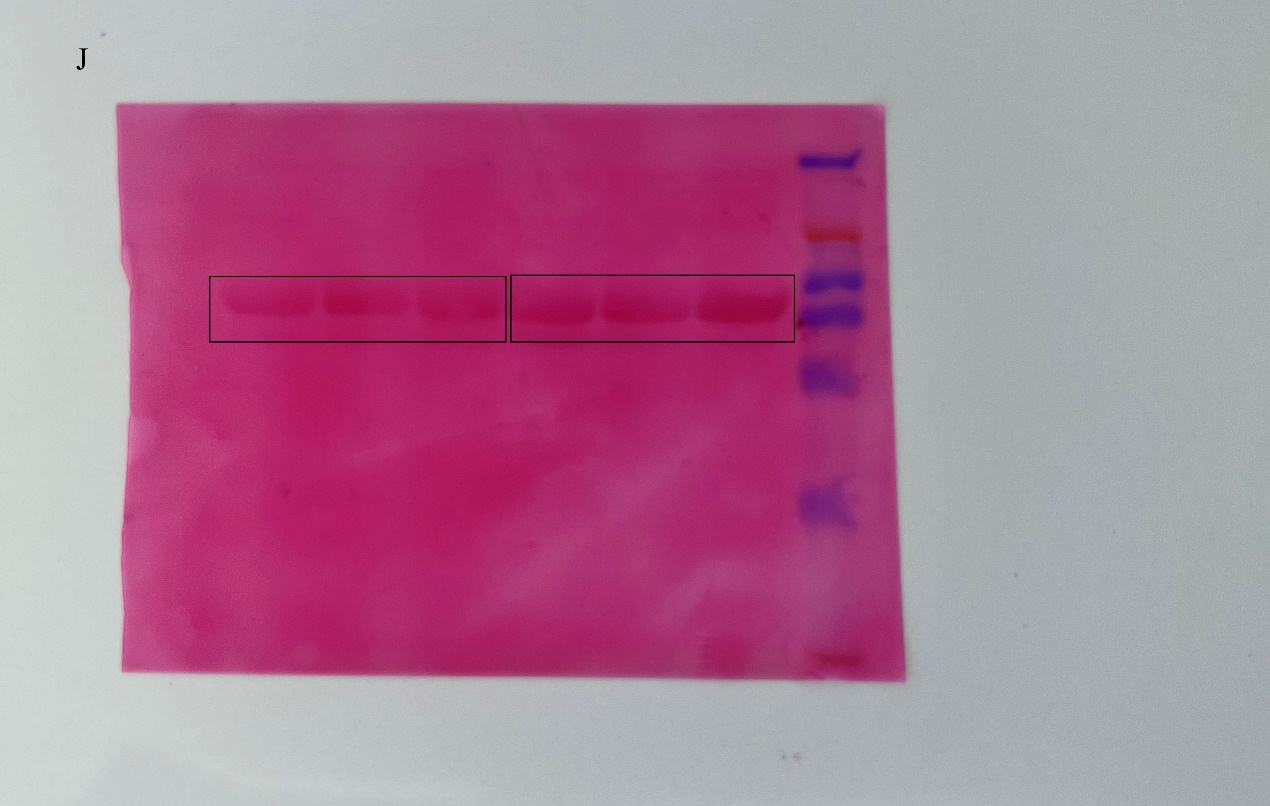


**Figure E-J: Supplement of** Figure 6B. Figure E: Inpute performed with anti-GFP (The black box indicates the boundary between the gels and rad box indicates the regions used in the main figures). Figure F: Ponceau S of Figure E (The black box indicates the regions used in the main figures). Figure G: Co-IP performed with anti-HA and the isolated protein was detected by Western blotting with an anti-GFP (The black box indicates the boundary between the gels and rad box indicates the regions used in the main figures). Figure H: Ponceau S of Figure G (The black box indicates the regions used in the main figures). Figure I: Inpute performed with anti-HA, Co-IP performed with anti-HA and the isolated protein was detected by Western blotting with an anti-HA (The black box indicates the boundary between the gels and rad box indicates the regions used in the main figures). Figure J: Ponceau S of Figure I (The black box indicates the regions used in the main figures).


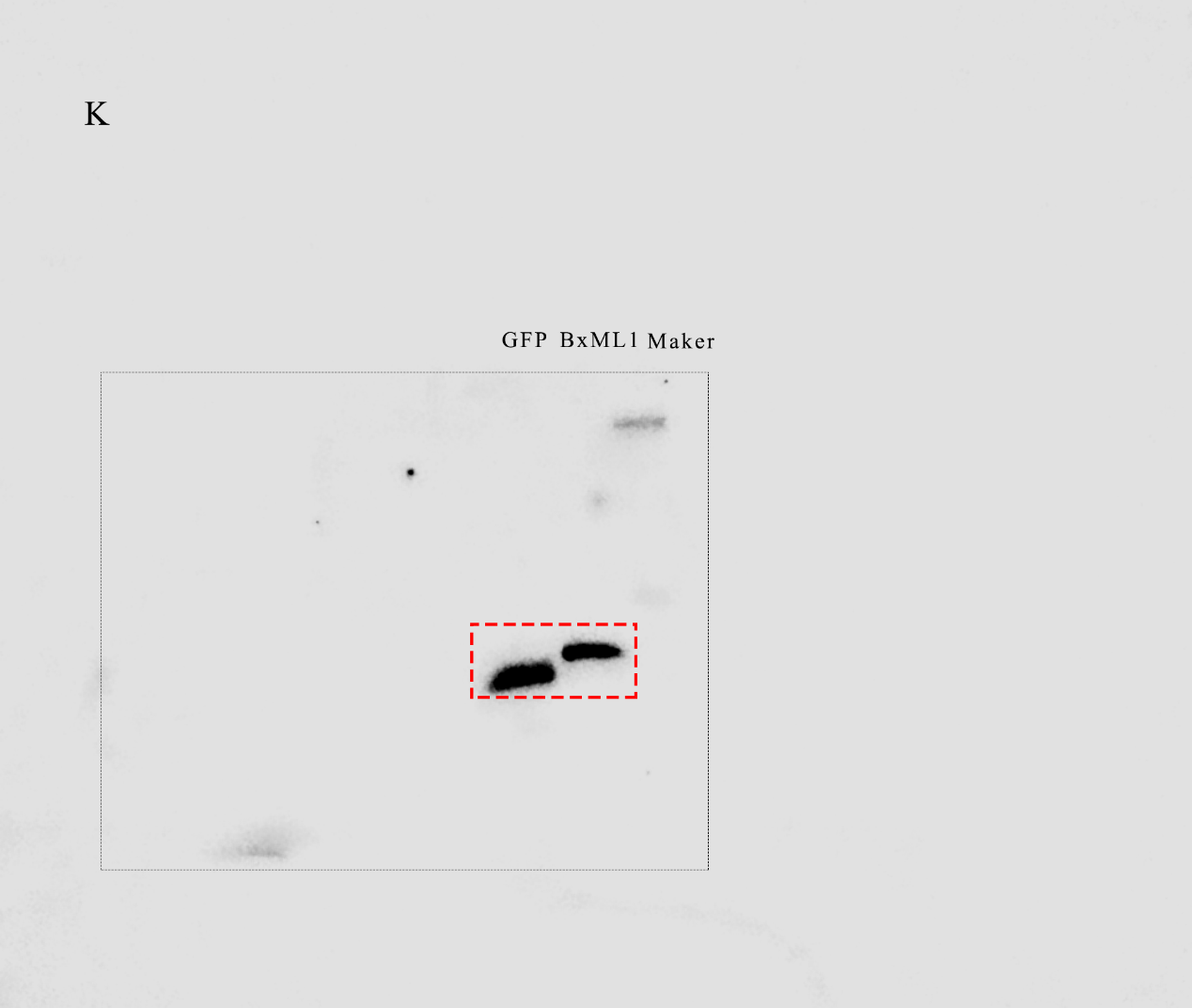


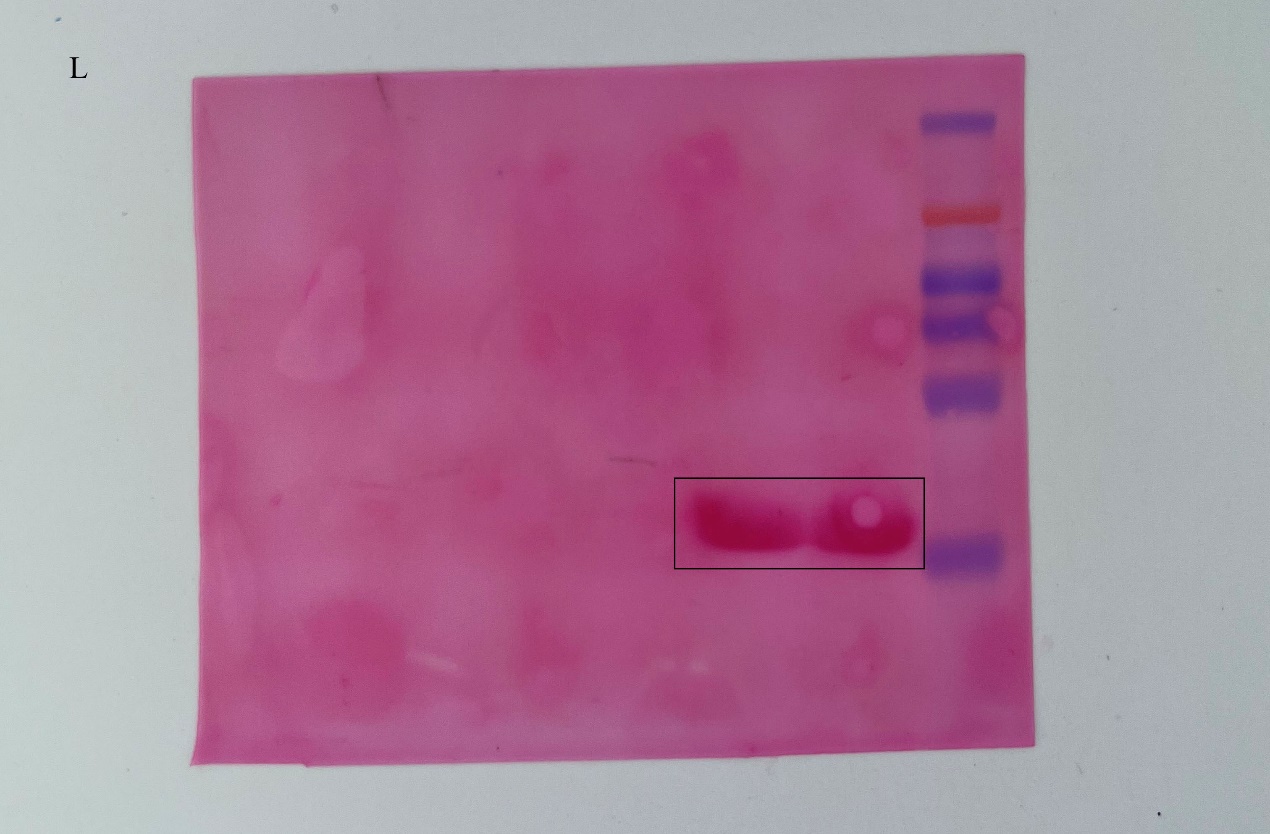


**Figure K L: Supplement of** Figure 7C. Figure K: Immunoblot analysis of purified BxML1 and GFP protein (The black box indicates the boundary between the gels and rad box indicates the regions used in the main figures). Figure L: Ponceau S of Figure K (The black box indicates the regions used in the main figures).


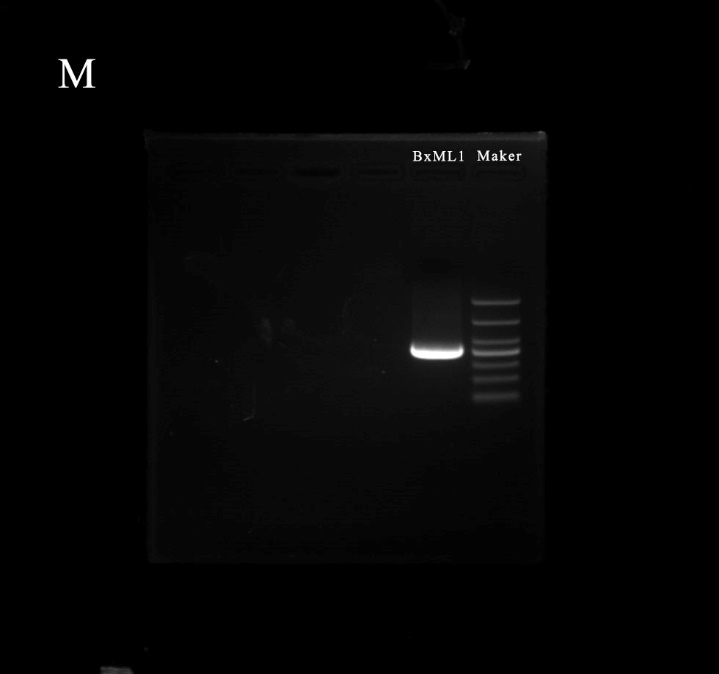


**Figure M: Supplement of** Figure S1A. Agarose gel electrophoresis of BxML1 gene.


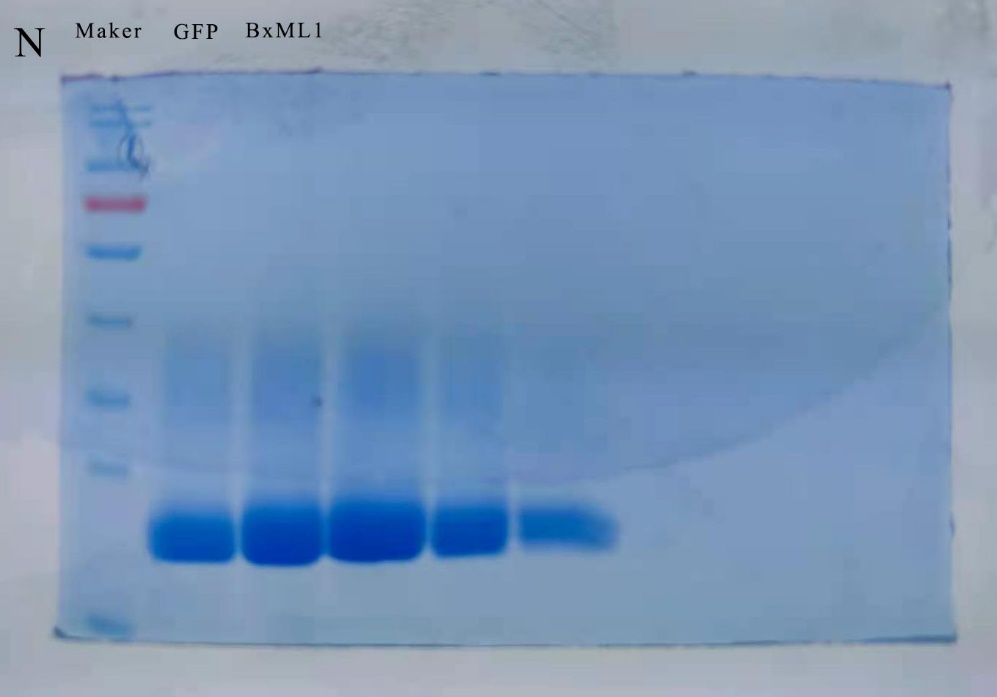


**Figure N: Supplement of** Figure S3. SDS gel of the purified BxML1 and GFP protein.
